# *Vibrio vulnificus* quorum-sensing molecule cyclo(Phe-Pro) inhibits RIG-I-mediated antiviral innate immunity

**DOI:** 10.1038/s41467-018-04075-1

**Published:** 2018-04-23

**Authors:** Wooseong Lee, Seung-Hoon Lee, Minwoo Kim, Jae-Su Moon, Geon-Woo Kim, Hae-Gwang Jung, In Hwang Kim, Ji Eun Oh, Hi Eun Jung, Heung Kyu Lee, Keun Bon Ku, Dae-Gyun Ahn, Seong-Jun Kim, Kun-Soo Kim, Jong-Won Oh

**Affiliations:** 1 Department of Biotechnology, Yonsei University, Seoul, 03722 Korea; 2 Department of Life Science, Sogang University, Seoul, 04107 Korea; 3 Graduate School of Medical Science and Engineering, Korea Advanced Institute of Science and Technology (KAIST), Daejeon, 34141 Korea; 4 Biomedical Science and Engineering Interdisciplinary Program, Korea Advanced Institute of Science and Technology (KAIST), Daejeon, 34141 Korea; 5 Center for Convergent Research of Emerging Virus Infection, Korea Research Institute of Chemical Technology, Daejeon, 34114 Korea

## Abstract

The recognition of pathogen-derived ligands by pattern recognition receptors activates the innate immune response, but the potential interaction of quorum-sensing (QS) signaling molecules with host anti-viral defenses remains largely unknown. Here we show that the *Vibrio vulnificus* QS molecule cyclo(Phe-Pro) (cFP) inhibits interferon (IFN)-β production by interfering with retinoic-acid-inducible gene-I (RIG-I) activation. Binding of cFP to the RIG-I 2CARD domain induces a conformational change in RIG-I, preventing the TRIM25-mediated ubiquitination to abrogate IFN production. cFP enhances susceptibility to hepatitis C virus (HCV), as well as Sendai and influenza viruses, each known to be sensed by RIG-I but did not affect the melanoma-differentiation-associated gene 5 (MDA5)-recognition of norovirus. Our results reveal an inter-kingdom network between bacteria, viruses and host that dysregulates host innate responses via a microbial quorum-sensing molecule modulating the response to viral infection.

## Abstract (web summary)

Quorum sensing signaling molecules are known to be critical determinants in bacterial pathogenesis. Here the authors show the quorum sensing molecule cFP from *Vibrio vulnificus* inhibits the RIG-I mediated antiviral interferon response and enhances susceptibility to viral infection.

## Introduction

Viral and microbial pathogen crosstalk may modulate disease outcomes of each pathogen by dysregulating host defense mechanisms. Diverse inter-pathogen crosstalk possibly occurs when two or more pathogens, which are either in the same or different kingdoms, co-infect the same host tissues or organs. In addition, even systemic inter-pathogen communication can be mediated by signal molecules released from individual pathogens or pathogen-infected cells when they circulate and deposit at remote sites where other pathogens propagate. Pathogen-associated molecular patterns (PAMPs) are known to be sensed by membrane-associated toll-like receptors (TLRs) or other receptors in the cytoplasm to initiate innate immune responses^1,2^. However, experimental evidence supporting virus-bacteria crosstalk through microbial quorum-sensing molecules is lacking^3,4^.

*Vibrio vulnificus*, which often flourishes in warm estuarine seawater or brackish environments, is an opportunistic pathogen causing fatal septicemia in humans upon entry by ingestion or wound infection, which results in up to 50% lethality^5^. Previous clinical studies and public health customs warn against raw oyster/shellfish consumption by patients with chronic liver diseases, since *V. vulnificus* contamination in such uncooked seafood can aggravate symptoms^6^. Particularly in immune-compromised people or those with liver diseases, such as cirrhosis or hepatitis, infection by this opportunistic pathogen is often associated with high lethality^7–10^. However, there is insufficient data regarding the molecular mechanisms of disease severity caused by co-infection of *V. vulnificus* and liver-specific viruses such as hepatitis C virus (HCV).

HCV, which has a positive-sense, uncapped RNA genome ~9.6-kb in length, is a leading cause of chronic liver disease, contributing to the development of liver cirrhosis and hepatocellular carcinoma^11^. Clinical case reports call for differential medical care of chronic HCV patients who are co-infected with *V. vulnificus*^8–10^. The severe disease outcomes caused by co-infection by these two pathogens prompted us to test the hypothesis that *V. vulnificus* might blunt antiviral innate immunity to HCV. As a potential messenger molecule that might be engaged in this inter-pathogen crosstalk, we focused on the *V. vulnificus*-produced quorum sensing molecule cyclo(Phe-Pro) (cFP) belonging to a family of cyclic dipeptides (CDPs) referred to as diketopiperazines (DKPs). CDPs, which are a group of hormone-like molecules synthesized by microbes as well as humans, are quorum sensing signal molecules that are used in cell–cell communication in bacteria^12–14^.

In this study, we elucidate the molecular mechanism of *V. vulnificus* cFP-mediated regulation of host innate immunity. We found that cFP inhibits retinoic acid-inducible gene-I (RIG-I) polyubiquitination, through its specific interaction with RIG-I, to blunt IRF-3 activation and type-I IFN production. We propose that a microbial quorum-sensing molecule-mediated tripartite virus-microbial pathogen–host interactions are important in determining host permissiveness to viral infection.

## Results

### The cyclic dipeptide cFP increases HCV loads

**Fig. 1:**
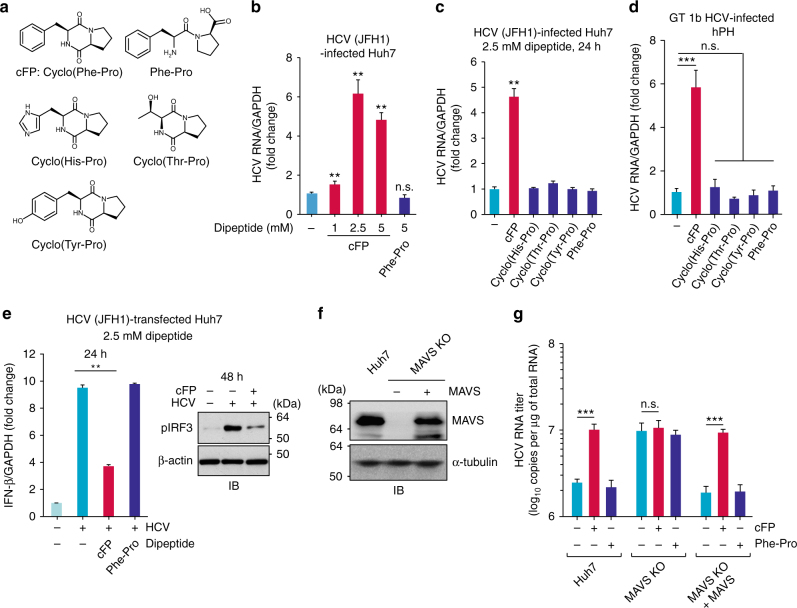
*V. vulnificus* cFP promotes HCV replication via dysregulation of IFN-β expression. **a** Molecular structures of various dipeptides used in this study. **b** RT-qPCR quantification of HCV genome levels in HCV-infected Huh7 cells treated with the indicated doses of cFP for 24 h. **c** Quantification of HCV RNA loads in HCV-infected Huh7 cells treated with 2.5 mM cFP or its derivatives for 24 h. **d** Quantification of HCV RNA loads in human primary hepatocytes treated with 2.5 mM cFP or its derivatives at 2 dpi with GT 1b HCV, which was derived from the humanized chimeric uPA-SCID mouse infected with HCV patient sera. **e** IFN-β mRNA and pIRF-3 expression levels in HCV (JFH1) RNA-transfected Huh7 cells one day after 2.5 mM cFP treatment, assessed by RT-qPCR and immunoblotting, respectively. **g** HCV (JFH1)-infected Huh7 or MAVS KO Huh7, which was transfected with an empty vector or a plasmid expressing Flag-tagged MAVS, was treated with 2.5 mM cFP or Phe-Pro for 48 h, prior to RT-qPCR quantification of HCV RNA. **f** Immunoblotting analysis for MAVS in Huh7 and MAVS KO Huh7, which were transfected as in **g**. In all panels, data are mean ± s.d. **P < 0.01; ***P < 0.001; n.s., not significant; by unpaired two-tailed Student’s *t*-test

We used cFP and its four different derivatives including the linear derivative Phe-Pro and structurally similar circular dipeptides such as cyclo(His-Pro), cyclo(Thr-Pro), and cyclo(Tyr-Pro) (Fig. 1a), to investigate their impact on HCV replication. Cyclo(His-Pro) is an endogenous cyclic dipeptide produced by the cleavage of the hypothalamic thyrotropin releasing hormone^15^. Cyclo(Tyr-Pro) is a quorum-sensing molecule involved in cell–cell communication by *Pseudomonas aeruginosa*^14,16^ and also acts as a signaling molecule regulating virulence gene expression in *Lactobacillus reuteri*^14,17^. All assays in the following experiments were performed at doses ≤5 mM at which cFP reduced cell viability <10% in HCV-permissive Huh7 cells (Supplementary Fig. 1a). We found that cFP significantly increases viral loads in HCV (JFH1, genotype 2a HCV)-infected Huh7 cells (Fig. 1b), upon entering into cells as verified by confocal microscopy using IAEDANS-labeled cFP and by uptake assays using [^14^C]cFP (Supplementary Fig. 1b–d). This enhancement was very specific to cFP; four other CDPs including the linear form of cFP, Phe-Pro did not modulate HCV RNA abundance (Fig. 1b, c). Similar results were also observed in genotype 1b (GT 1b) HCV-infected human primary hepatocytes (Fig. 1d).

Having found HCV-promoting activity of cFP, we investigated whether it interferes with interferon production. IFN-β mRNA expression was significantly increased in JFH1 RNA-transfected Huh7 cells (Fig. 1e; 9.7-fold increase over untreated control, **P < 0.01 by unpaired Student’s *t*-test). This IFN-β mRNA upregulation was suppressed specifically by cFP. Interferon regulatory factor 3 (IRF-3) activation induced by JFH1 RNA transfection was also repressed by cFP as assessed by immunoblotting for pIRF-3.

To understand how cFP abrogates the IFN production signaling pathway cascade to promote HCV infection, we first investigated whether activation of the mitochondrial antiviral signaling protein (MAVS; also called IPS-1, CARDIF, or VISA) downstream effector TANK-binding kinase 1 (TBK1) is affected. TBK1 phosphorylation at Ser172 within its activation loop is required for its kinase activity responsible for phosphorylation of its downstream substrate IRF-3^18,19^. An in vitro kinase assay using immunoprecipitated TBK1 showed that its activity is not directly inhibited by cFP, even at millimolar concentrations (Supplementary Fig. 1e).

The above results prompted us to assess the implication of MAVS for cFP-induced HCV replication stimulation. In a MAVS knockout (KO) Huh7 cell line established by the CRISPR-CAS9 gene-editing system (Supplementary Fig. 2), the HCV regulatory activity of cFP disappeared (Fig. 1f, g). Furthermore, rescue of MAVS expression by transfection of a MAVS-encoding plasmid restored the sensitivity to cFP in HCV-infected MAVS KO Huh7 cells, while Phe-Pro had no influence on HCV loads regardless of MAVS expression (Fig. 1f). We further investigated the effect of cFP in primary hepatocytes isolated from MAVS-deficient (*MAVS*^−/−^) mice^20^. Because HCV does not infect mouse cells due to the lack of human homology receptors^21^, we carried out a transient HCV RNA transfection experiment. As assessed by confocal microscopy, cFP failed to interfere with HCV gene expression in primary hepatocytes isolated from *MAVS*^−/−^ mice; GFP reporter (as a NS5A-GFP fusion protein) expression was not altered significantly in cells transfected with JFH1/GFP RNA, a JFH1 derivative capable of expressing the NS5A-GFP fusion protein (Supplementary Fig. 3a, b). In parallel experiments using normal mouse (BALB/c) primary hepatocytes, cFP, but not Phe-Pro, increased NS5A-GFP expression (Supplementary Fig. 3c, d). As expected, IFN-β mRNA expression induced by HCV RNA was also significantly suppressed by cFP (Supplementary Fig. 3e) as observed in the HCC cell line Huh7.

Together, our data suggested that cFP might target MAVS upstream signaling molecules such as the well characterized cytoplasmic foreign RNA-sensing pattern recognition receptors (PRRs), RIG-I and MDA5. Because mouse hepatocytes support only one-step replication due to the lack of human-compatible receptors for HCV entry^21^, these results also suggested that cFP regulates post-entry replication steps.

### RIG-I is a specific target of cFP

**Fig. 2:**
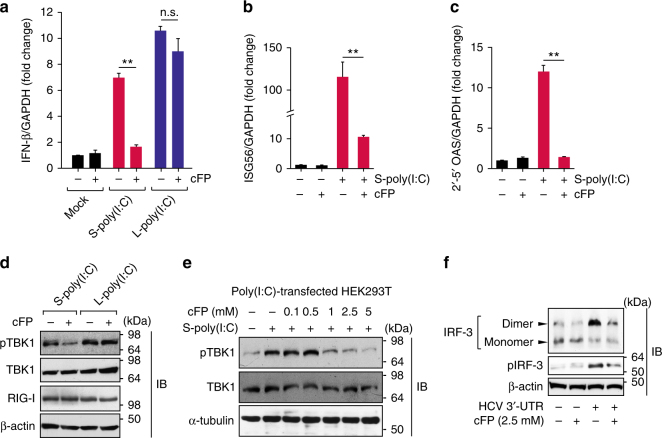
cFP inhibits RIG-I-mediated innate immune responses. **a**–**d** HEK293T cells, which were stimulated with 2 μg ml^−1^ of indicated poly(I:C), were left untreated or treated with 2.5 mM cFP for 12 h, prior to quantification of IFN-β (**a**), ISG56 (**b**), and 2′-5′ OAS (**c**) mRNA levels or immunoblotting analysis for the indicated proteins (**d**). In **a**–**c**, data are mean ± s.d. **P ≤ 0.01; n.s., not significant; by unpaired two-tailed Student’s *t*-test. **e** Inhibition of TBK1 activation by cFP. Activated TBK1 (pTBK1) levels in HEK293T cells transfected with 1 μg ml^−1^ of S-poly(I:C) were monitored by immunoblotting 6 h after cFP treatment. **f** Analysis of IRF-3 and its activated form (pIRF-3) expression in HCV 3′-UTR RNA-transfected HEK293T cells, which were left untreated or treated with 2.5 mM cFP. Cell lysates were subjected to native PAGE or SDS-PAGE for immunoblotting analysis for IRF3 dimers and pIRF-3, respectively at 12 h post-transfection with HCV 3′-UTR

RIG-I and MDA5 sense respectively the PAMPs within viral RNA and viral RNA replication products including the dsRNA replication intermediates produced by RNA viruses^22^. To discern which of these sensor molecules is the cFP target, we used two different forms poly(I:C) (a synthetic double-stranded RNA mimic), a short-length form [<0.3-kb; S-poly(I:C)] and an extended-length form [>5-kb; L-poly(I:C)] (Supplementary Fig. 4a), which were recognized by RIG-I and MDA5, respectively, to induce IFN-β mRNA expression via the MAVS (Supplementary Fig. 4b). These results were consistent with previous studies^23^. Surprisingly, we found that cFP selectively interferes with S-poly(I:C)-triggered IFN-β production (Fig. 2a). Furthermore, expression of the interferon-stimulated genes ISG56 and 2′-5′ OAS, which was induced by S-poly(I:C), was also reduced upon cFP treatment (Fig. 2b, c). Notably, cFP per se did not alter these IFN-inducible gene expression levels, suggesting its incapacity to modulate non-activated RIG-I function. Importantly, TBK1 activation triggered by S-poly(I:C) was only selectively repressed dose dependently by cFP, while L-poly(I:C)-induced TBK1 activation pathway was not influenced by cFP (Fig. 2d, e). These results suggested that the potential target of cFP is the RIG-I signaling pathway.

RIG-I senses an RNA ligand carrying a 5′-triphosphate moiety, which is an important molecular signature for RIG-I to recognize virally-produced non-self RNA molecules. In addition to this, the poly-U/UC sequence within the HCV 3′-UTR was also identified as a PAMP motif^24,25^. As expected, HCV 3′-UTR induced IRF-3 activation, as evidenced by IRF-3 phosphorylation and dimerization (Fig. 2f). This IRF-3 activation was effectively blocked by cFP in HEK293T cells. Overall, our results demonstrate that cFP interferes with RIG-I activation.

In addition to the PRR-MAVS axis, TBK1 activation can occur through the TLR4-TRIF axis^26–28^. We found that TBK1 activation induced by LPS, a prototype TLR4 ligand, was not regulated by cFP in primary hepatocytes from *MAVS*^−/−^ mice (Supplementary Fig. 5), demonstrating that TBK1 in the TLR-TRIF axis is not affected by cFP. This result is consistent with our results showing that TBK activity is not directly inhibited by cFP (Supplementary Fig. 1e).

### cFP binding to RIG-I blunts the host innate immune response

**Fig. 3:**
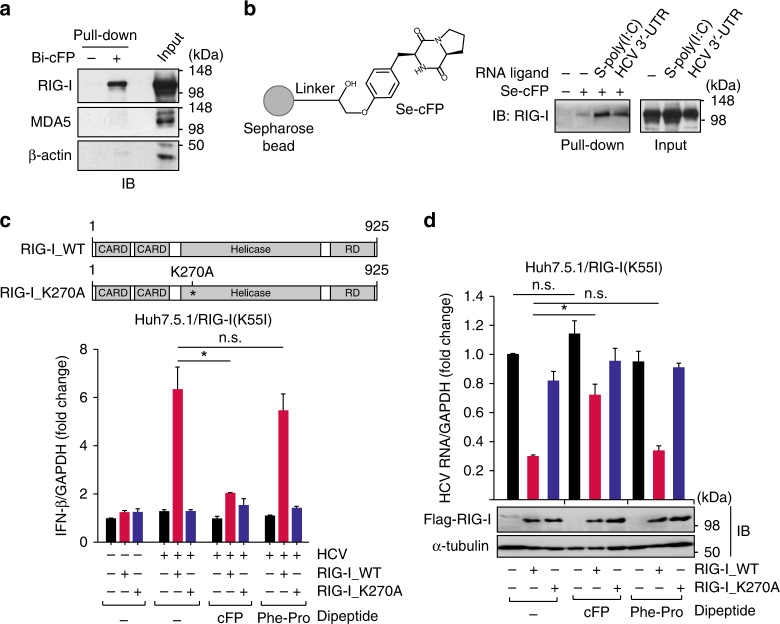
HCV-promoting activity of cFP is mediated through its specific interaction with RIG-I. **a**, **b** Immunoblotting analysis of cFP-interacting proteins pull-downed by biotinylated cFP bound to Streptavidin beads (**a**) or by non-modified cFP immobilized onto Sepharose beads (**b**). HEK293T cells, which were non-stimulated (**a**) or stimulated with the indicated RNA ligands (**b**) were used in pull-down experiments. **c**, **d** Quantification of IFN-β mRNA (**c**) and HCV genome (**d**) levels in Huh7.5.1 cells transfected with an empty vector or a vector expressing the wild-type RIG-I (RIG-I_WT) or its inactive mutant (RIG-I_K270A). After 18 h, the transfected cells were infected with HCV (JFH1) by incubation for 6 h and incubated further for 24 h in fresh media without or with the indicated peptides (2.5 mM) prior to RT-qPCR analyses. Statistical significance of differences between groups was determined via unpaired two-tailed *t*-test. *P ≤ 0.05; n.s., not significant

We sought to test if cFP binds to RIG-I to inhibit RIG-I-dependent type I IFN production. Protein complex pull-down experiments using biotinylated cFP revealed that cFP indeed interacts with RIG-I, but not MDA5 (Fig. 3a). These results are consistent with our finding that cFP does not interfere with the MDA5 signaling pathway triggered by L-poly(I:C) (Fig. 2d) and suggested that cFP binds to RIG-I with a certain specificity. Using cFP-conjugated Sepharose beads, we further verified the interaction between cFP and RIG-I (Fig. 3b). This interaction occurred both in non-stimulated cells and in cells stimulated with either S-poly(I:C) or HCV 3′-UTR, with the interaction being noticeably enhanced when RIG-I was activated with its ligands. This suggested that cFP-binding to RIG-I might be facilitated upon its activation through RNA ligand binding.

To provide direct evidence for functional interaction between cFP and RIG-I, we took advantage of the Huh7.5.1 cell line^29^ that expresses a defective RIG-I with a T55I substitution^30^. This mutant RIG-I was shown to be incapable of transmitting signals through its N-terminal CARD domain while its ability to sense RNA ligands was not affected^31^. When this cell line was transfected with HCV RNA, there was no substantial induction of IFN-β mRNA expression as expected, while ectopic expression of wild-type RIG-I, but not its inactive mutant (RIG-I_K270A) carrying a mutation at the ATP-binding site, rescued IFN-β mRNA expression, which could be then reversed specifically by cFP (Fig. 3c). HCV load reduction caused by ectopic RIG-I expression in Huh7.5.1 cells was also specifically reversed by cFP treatment (Fig. 3d). These results strongly demonstrate RIG-I involvement in cFP-mediated dysregulation of IFN-β production. In addition, depletion of RIG-I by RNAi resulted in blunting the HCV regulatory activity of cFP in HCV-infected Huh7 cells, reinforcing the inhibitory role of cFP in sensing of HCV RNA by RIG-I (Supplementary Fig. 6).

**Fig. 4:**
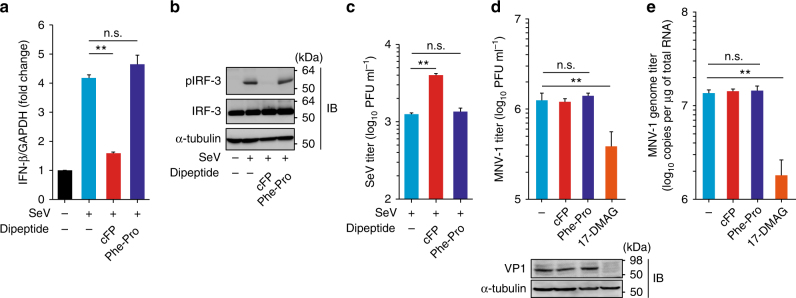
Differential effect of cFP on SeV and norovirus replication. **a**–**c** HEK293T cells infected with SeV were treated with 2.5 mM cFP or Phe-Pro. After 24 h, cells were harvested to assess IFN-β mRNA levels (**a**), IRF-3 activation (**b**), and infectious virus titers in culture media (**c**). **d**, **e** Mouse macrophage cells (RAW264.7), which were infected with mouse norovirus (MNV-1) at an MOI of 0.005, were treated with 2.5 mM cFP or Phe-Pro. 17-DMAG (300 nM), an Hsp90 inhibitor known as an anti-norovirus reagent, served as a positive control. After 24 h, infectious virus titer in culture media and intracellular viral genome levels were determined by a plaque-forming assay (**d**) and RT-qPCR (**e**), respectively. Immunoblot for the MNV-1 VP1 is shown below the bar graph in **d**. **P < 0.01; n.s., not significant; by unpaired two-tailed Student’s *t*-test

With Sendai virus (SeV) well known to generate RIG-I ligands^32^, we were able to further confirm cFP’s ability to inhibit IFN-β mRNA expression and IRF-3 activation induced by the viral infection (Fig. 4a, b). cFP treatment consequently resulted in a significant increase in infectious SeV titer (Fig. 4c). In contrast, norovirus infection, which was previously reported to trigger MDA5-mediated type-I IFN production in dendritic cells and in mice^33^, was not affected by cFP treatment (Fig. 4d, e). These results collectively support our conclusion that cFP specifically targets the RIG-I pathway.

### cFP inhibits TRIM25-meidated RIG-I ubiquitination

**Fig. 5:**
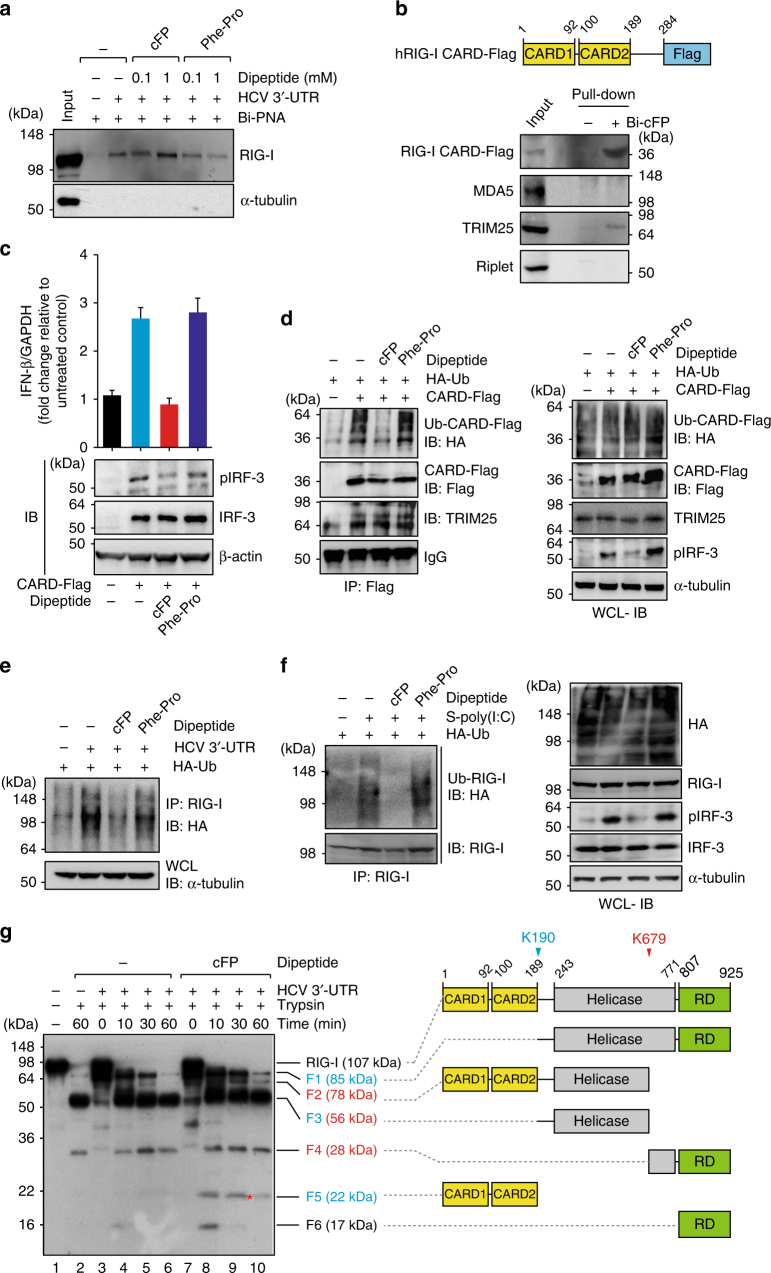
cFP binding to the RIG-I CARD domain induces a conformational change to hinder its ubiquitination. **a** Neither cFP nor Phe-Pro interferes with RNA sensing by RIG-I. Immunoblotting analysis for RIG-I in the protein complex pull-downed by HCV 3′-UTR, which was immobilized onto Streptavidin beads via the 17-mer biotinylated PNA-SL3-17 (Bi-PNA) targeting HCV 3′-end X-RNA stem-loop III region, in the presence of the indicated peptides. **b** A pull-down experiment was performed as described in Fig. 3a, with non-stimulated HEK293T cells that transiently expressed the Flag-tagged RIG-I CARD region. **c**,** d** HEK293T cells, which transiently expressed Flag-tagged RIG-I CARD region without (**c**) or with an HA-tagged ubiquitin (HA-Ub) (**d**), were left untreated or treated with cFP or Phe-Pro, prior to quantification of IFN-β mRNA levels (**c**) and immunoblotting analysis for the indicated proteins (**c**,** d**). Shown on the right panel in **d** are immunoblots of whole cell lysates (WCL). **e**,** f** HEK293T cells, which transiently expressed an HA-tagged ubiquitin for 12 h, were stimulated by intracellular delivery of HCV 3′-UTR RNA (1 μg ml^−1^) (**e**) or S-poly(I:C) (**f**) by liposome-mediated transfection and further incubated with 2.5 mM cFP for 6 h, prior to immunoblotting analysis for the indicated proteins as in **d**. **g** Limited trypsin digestion of RIG-I was performed in the presence of HCV 3′-UTR RNA for the indicated reaction times. Cleaved fragments of RIG-I were subjected to SDS-PAGE and visualized by immunoblotting with polyclonal antibody against full-length recombinant human RIG-I. Predicted molecular masses of cleaved RIG-I fragments [amino acid positions are numbered according to the GenBank sequence (NP_055129.2)] are shown

Having identified the target of cFP, we asked how RIG-I function becomes impaired upon cFP binding. RIG-I has tandem N-terminal caspase recruitment domains (CARDs), a DExD/H-box helicase, and a C-terminal domain (CTD). Short dsRNA with a 5′ triphosphate moiety is sensed by the CTD and helicase domain^34,35^. Pull-down experiments using in vitro transcripts HCV 3′-UTR immobilized on beads revealed that cFP as well as Phe-Pro does not alter RIG-I’s ability to sense its target RNA (Fig. 5a). We next assessed the possibility of interaction between cFP and the N-terminal 2CARD region by a pull-down assay using immobilized biotinylated cFP. As shown in Fig. 5b, cFP specifically interacted with 2CARD region of RIG-I; MDA5 with a homologous CARD domain was not detected (Fig. 5b). Both TRIM25, which interacts with the N-terminal CARDs of RIG-I, and Riplet, which binds to the RIG-I C-terminal repressor domain (RD), are known to mediate the K63-linked ubiquitination of RIG-I to induce IFN-β production^36–38^. As expected, TRIM25, but not Riplet, was found to be associated with the ectopically expressed CARD-Flag, which was consistent with the established notion that its recruitment for RIG-I ubiquitination needs to be preceded by CARD domain release, which occurs following RNA ligand sensing. More importantly, IFN-β mRNA expression and IRF-3 activation, which were induced by overexpression of RIG-I CARD domain, were suppressed by cFP (Fig. 5c), identifying the N-terminal CARD domain of RIG-I as the cFP target. Using the mouse CARD domain, which is highly homologous (77% amino acid sequence identity) to human RIG-I CARD domain (Supplementary Fig. 7a), similar IFN-β induction inhibitory activity of cFP was observed (Supplementary Fig. 7b), demonstrating that cFP-mediated RIG-I dysregulation is a common feature shared by murine and human cells.

Polyubiquitination of the RIG-I CARD domain, which is released upon RNA ligand binding, is required for activation of the IFN signaling pathway by facilitating its interaction with the CARD domain of MAVS^39^. As shown in Fig. 5d, RIG-I activation, as evidenced by its polyubiquitination, was specifically blocked by cFP in HCV 3′-UTR-stimulated cells. Furthermore, IRF-3 activation, as assessed by pIRF-3 immunoblotting, was also suppressed by cFP. Altogether, these results suggest that cFP binds to the 2CARD region without blocking TRIM25 binding and interferes with RIG-I activation by inhibiting its TRIM25-mediated ubiquitination. We could observe similar inhibition of endogenous RIG-I polyubiquitination by cFP in cells treated with HCV 3′-UTR (Fig. 5e), as well as in cells stimulated with S-poly(I:C) (Fig. 5f). Furthermore, IRF-3 activation induced by S-poly(I:C), was also suppressed by cFP. Altogether, these results further prove an inhibitory role for cFP in RIG-I ubiquitination-mediated activation.

Previously, RIG-I was shown to be cleaved at K190 and K679 residues by limited trypsin digestion to produce diverse digested products including the ~22-kDa 2CARD region (F5 fragment in Fig. 5g), ~56-kDa helicase region (F3 fragment), and ~28-kDa C-terminal region including the repressor domain (RD) (F4 fragment)^40^. We detected two major digestion products (F3 and F4 fragments) accumulated after 60 min trypsin digestion in the absence or presence of HCV 3′-UTR (compare lanes 2 and 6). Thus, it appears that both the K190 and K679 sites are exposed to trypsin regardless of the presence of HCV 3′-UTR. Notably, the 2CARD (F5 fragment) was barely detectable under these digestion conditions when cFP was not added to the assay mixture (lanes 2–6). In a parallel experiment conducted in the presence of cFP, this trypsin vulnerable fragment, which started to accumulate after 10 min digestion, still remained detectable at 60 min (lanes 8–10), suggesting cFP binding to this 2CARD region might induce a conformation change to confer resistance to trypsin digestion.

### In vivo regulation of IFN-β production by cFP

**Fig. 6:**
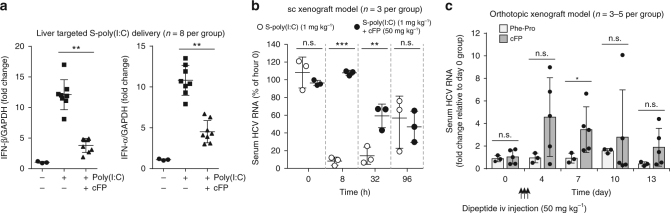
Suppression of type I IFN production by cFP and its impact on HCV replication in mice. **a** cFP in 100-μl saline was injected into tail veins of BALB/c mice (8 weeks old, *n* = 8) at a dose of 50 mg cFP per kg of body weight. After 1 h, poly(I:C) formulated in lipidoid nanoparticles (100 μl) was administered through the tail vein at a dose of 1 mg per kg body weight. After 8 h stimulation, IFN-α/β mRNA levels were analyzed using total RNA extracted from the large lobe of mouse liver. **b**, **c** Quantification of serum HCV RNA titers in mouse models of HCV replication. In **b**, NOD/SCID mice carrying s.c. HCV-infected Huh7 xenografts (9 weeks old, *n* = 3 per group) were pre-treated with cFP for 1 h prior to poly(I:C) stimulation, as described in **a**. In **c**, an orthotopic mouse model with a chimeric liver transplanted with HCV-infected Huh7 was used (9 weeks old, *n* = 3–5 per group). After three consecutive cFP or Phe-Pro injections (50 mg per kg body weight once a day for 3 days) starting on day 1, serum HCV RNA loads were monitored. All data were presented as a fold-change relative to the groups which received Phe-Pro. In all panels, error bars are standard deviations. In **a**, data are from three independent experiments, each involving triplicate assays. Statistical significance of differences between groups was determined via unpaired two-tailed *t*-test (**b**) or Welch’s *t*-test (**c**). *P < 0.05; **P < 0.01; ***P < 0.001; n.s., not significant

We next investigated in mice the regulatory role of cFP in IFN-β production. cFP delivered systematically via i.v. injection through the tail vein suppressed IFN-α/β mRNA expression that was induced by priming with an i.v. injection of S-poly(I:C) (Fig. 6a). In a mouse model carrying HCV-infected Huh7 subcutaneous (s.c.) xenografts, which was established, as described previously^41^, i.v. injection of poly(I:C) led to a substantial reduction in serum HCV genome titer 8 h after poly(I:C) stimulation. This inhibitory effect was completely reversed by administration of cFP (Fig. 6b). The HCV promoting activity of cFP gradually diminished thereafter, disappearing three days later. In an orthotopic mouse model carrying HCV-infected Huh7 cells transplanted into the liver^42^, cFP administration (once each day at a dose of 50 mg per kg body weight for a total of three consecutive days) increased serum HCV loads substantially four and seven days after the first cFP administration, which gradually declined to show non-significant difference by days 10 and 13 compared with the control group that received Phe-Pro (Fig. 6c).

**Fig. 7:**
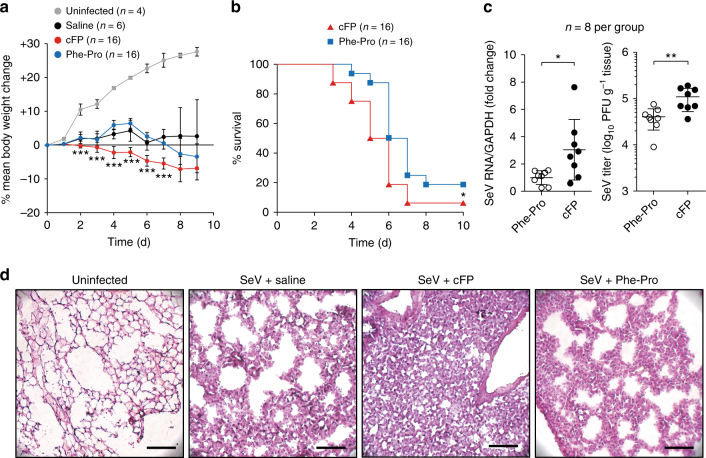
Increase of mortality of Sendai virus-infected mice by cFP administration. DBA/2 mice (6-week-old male), which were given cFP or Phe-Pro (50 mg per kg body weight) by i.v. injection, were infected with Sendai virus (1,000 PFU) by intranasal injection 2 h after the dipeptide administration followed by two additional consecutive administrations at 1 and 2 days post-infection, prior to monitoring body weight change (**a**) and mouse mortality (**b**) over 10 days. Virus titers were determined by RT-qPCR (for RNA genome loads) and plaque-forming assays at 7 dpi (**c**). Shown in **d** are representative H&E stain images of lung sections at 7 dpi. Scale bar=200 μm. Statistical significance of differences between cFP-treated group and vehicle-treated, uninfected control group was determined via unpaired two-tailed *t*-test. Survival curves were analyzed using log rank (Mantel-Cox) test. *P < 0.05; **P < 0.01, ***P < 0.001

RIG-I-specific regulatory functions of cFP were further verified in mice infected with SeV and influenza virus, which were previously shown to induce immune responses through RIG-I^32,39,43^. cFP administration via i.v. injection increased mortality in mice infected SeV (Fig. 7a, b) In consistent with the in vitro results observed in SeV-infected cells (Fig. 4a–c), virus titers in lung tissues were increased 3-fold and 2.7-fold, as assessed by RT-qPCR and plaque-forming assays, respectively, in mice that received cFP (Fig. 7c). Histological analysis of lung tissue sections revealed that influenza infection-mediated lung injury, which was associated with immune cell infiltration into the lung and alveolar destruction, became more severe in mice receiving cFP at 7 days post-infection (dpi) (Fig. 7d). Similarly, cFP significantly increased mortality in mice infected with influenza A virus (PR8 strain) (Supplementary Fig. 8a, b). As in SeV-infected mice, alveolar destruction was intensified by cFP in the influenza-infected mice (Supplementary Fig. 8c). These results together demonstrate that cFP is a specific regulator inhibiting the RIG-I pathway.

## Discussion

A number of previous studies have enhanced our understanding of how PAMPs mount innate immune responses and how pathogens use diverse strategies to block the sensing of those non-self patterns by diverse PRRs. However, inter-pathogen crosstalk through molecules produced by each pathogen in innate immune responses has been less studied despite the potential importance of such crosstalk in determining disease outcomes of each pathogen upon co-infection. One particular regulatory network that has been little-characterized in the virus-microbial pathogen-host tripartite interplay is an indirect control mechanism for viral replication by a microbial quorum sensing molecule. In the present work, we demonstrated that *V. vulnificus*-produced cFP is a microbial ligand that increases HCV loads in cell culture and in mouse models of HCV replication.

**Fig. 8:**
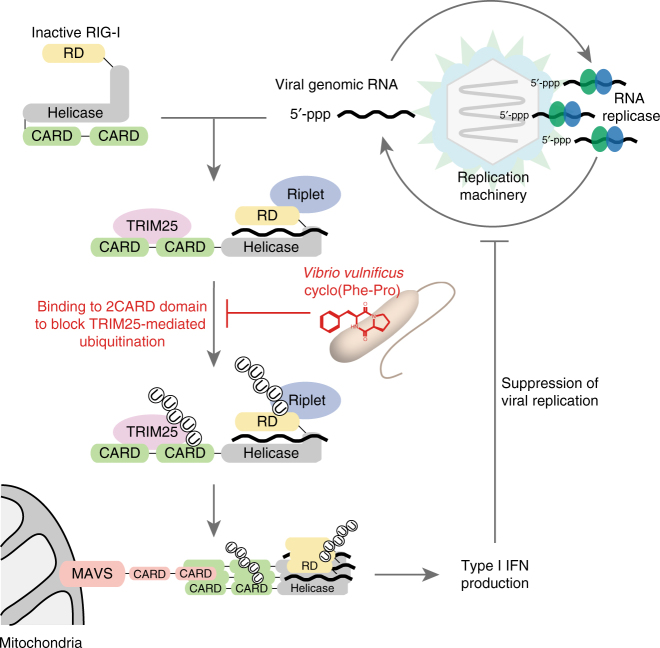
Model for *V. vulnificus* cFP-mediated dysregulation of host innate immune responses. cFP promotes propagation of RNA viruses that are sensed by RIG-I, but not the ones inducing type-I IFN production through MDA5. It binds to the RIG-I CARD domain and induces a conformational change to block RIG-I polyubiquitination essential for induction of antiviral innate immune responses. The inter-pathogen crosstalk mediated through a quorum sensing molecule from pathogenic microbes may play an important role in determining the outcome of virus–host interactions

The HCV-promoting activity of cFP was attributed to its ability to block RIG-I-mediated type I IFN production. The molecular mechanism behind this regulation seems to be related to a RIG-I conformational change upon cFP binding to the 2CARD region, resulting in abrogation of TRIM25-mediated RIG-I polyubiquitination essential for its activation and signal transmission to the MAVS through interactions between CARD domains of RIG-I and MAVS (Fig. 8). Importantly, we demonstrated that, in addition to HCV, SeV and influenza virus, which were previously known to be sensed by RIG-I^39^, were affected by cFP. MDA5^33^, which senses dsRNA >2-kb independently of its 5′-end modification state^23^, was not functionally regulated by cFP. Thus, we propose that RIG-I, which recognizes cytoplasmic, non-self RNAs carrying a terminal 5′-triphosphate and also a set of small dsRNAs^39^, is the primary target of cFP. This selective inhibitory activity of cFP toward RIG-I is likely due to the fact that the N-terminal CARD domains of RIG-I and MDA5 display only 25% amino acid identity, although they are functionally similar each other^44^. Furthermore, RIG-I pathway specific inhibition by cFP is explained by the fact that the 2CARDs of RIG-I, but not MAD5, exclusively undergoes ubiquitination^36^. Gack et al.^31^ showed that the T55 residue of the RIG-I first CARD is critical for TRIM25 binding. Because cFP bounds to the 2CARD without blocking its interaction with TRIM25, it is very likely that cFP targets the second CARD where TRIM25-mediated ubiquitination occurs at the K172 residue.

What might be the impact of cFP in normal host cells? Our results showed that cFP per se does not enhance IFN-inducible gene expression at least in non-infected cells. The inhibitory activity of cFP disappeared in the Huh7.5.1 cell line in which TRIM25 cannot bind to the RIG-I due to a T55I mutation^31^. These findings strongly suggest that cFP exclusively dysregulates activated RIG-I by inhibiting its TRIM25-mediated ubiquitination. Accordingly, it may exert its negative regulatory role not only in virus-infected cells but also in in cells in which RIG-I is aberrantly activated. The helicase activity of RIG-I is indispensable in a dsRNA-induced antiviral response^45^. Recent studies proposed that the helicase or ATPase activity is also important in imparting RIG-I’s self-RNA discrimination nature: rapid release of self-RNAs, which might occur due to robust helicase activity was elucidated as a mechanism for preventing aberrant IFN production by self-RNAs^46–48^. Mutation of RIG-I with an E373A substitution in the hel1 motif of the RIG-I helicase domain was reported to result in constitutive activation of RIG-I, leading to aberrant production of IFN in Singleton–Merten syndrome patients^46,49^. cFP is not likely to inhibit RIG-I helicase activity since IFN-β expression was not induced by cFP. Because cFP binds to the RIG-I regardless of its association with RNA ligands, cFP may prevent aberrant non-self RNA-mediated stimulation of IFN responses by blocking RIG-I activation. Thus, it is tempting to speculate that cFP might be used to cure the aforementioned genetically-determined inflammatory disorder by blocking polyubiquitination-mediated RIG-I activation.

Regarding the severe liver dysfunction observed in chronic HCV patients co-infected with *V. vulnificus*^7–10^, several possible interpretations other than cFP-mediated inhibition of RIG-I activation may also explain such fulminant liver disease with high mortality. Iron-overloaded mice were shown to be more susceptible to *V. vulnificus* infection^50^. Because increased hepatic iron content is a common finding among patients with chronic HCV infection^51^, homing of *Vibrio* to the liver may aggravate chronic HCV infection. *V. vulnificus* PAMPs^7–10^, which can stimulate multiple TLRs in the liver, may also lead to liver failure^52,53^. For instance, production of proinflammatory cytokines induced by *V. vulnificus* virulence factors such as capsular polysaccharide, lipopolysaccharide, cytotoxins, pili, and flagellum may explain the vulnerability of patients affected by HCV related-chronic liver disease^9,54^. Thus, we cannot rule out the *V. vulnificus* infection-associated sepsis as a cause of the liver dysfunction. It is also important to note that the susceptibility to *V. vulnificus* sepsis is influenced by predisposing conditions, such as viral hepatitis, immune compromise, and cirrhosis^55^, which may explain its correlation with risk factors in predisposed individuals. However, the nature of such factors in high-risk populations including chronic HCV patients remain unknown. Although it is thus challenging to estimate relative contribution of cFP to the severe liver dysfunction during acute infection of *V. vulnificus*. Our results raise a possibility that its quorum-sensing molecule cFP targets RIG-I to impair host innate immune response, resulting in promotion of HCV propagation. Our data also showed that circulating cFP aggravates pathogenesis of respiratory tract-infecting viruses such as influenza virus and Sendai virus. Thus, attenuating RIG-I-mediated immune responses by cFP is not restricted to HCV.

Since DKPs are produced by various microbial cells, as well as by other organisms, including marine sponges and animals^56^, a more broad question that needs to be addressed is whether other microbial quorum-sensing dipeptide molecules also impact on innate immunity. Among the DKPs tested in this study, only cFP specifically regulated the RIG-I pathway. Therefore, what remains to be studied is whether other known microbial quorum sensing molecules suppress or augment host innate immune responses. Recently, gut microbiome (population diversity and abundance of each member of the microbial community) has also been emphasized as an important modulator controlling the progress of diverse diseases^57^. Thus, it would be of interest to investigate whether gut microbiomes as well as enteric bacterial pathogens regulate viral diseases through their secreted quorum-sensing molecules by dysregulating RIG-I or other PRRs.

In summary, we demonstrated that *V. vulnificus* cFP blunts the establishment of RIG-I-mediated antiviral resistance, providing an elegant example of a microbial quorum sensing molecule dysregulating host innate immunity. Our results pave a new avenue to the understanding of inter-pathogen interplays that might determine the outcomes of diseases developed by single infection by each pathogen. Further studies are warranted to understand pathophysiological functions of other bacterial quorum sensing molecules in the context of tripartite (host–virus–microbe) interactions.

## Methods

### Reagents

Cyclo(L-Phe-L-Pro) (cFP) and Phe-Pro were purchased from Bachem (Bubendorf, Switzerland) and Sigma-Aldrich (St. Louis, MO, USA), respectively. Other cyclic dipeptides including biotinylated cFP and IAEDANS-labeled cFP were synthesized in-house at Sogang University, Seoul Korea. An anti-norovirus reagent 17-DMAG^58^, *E. coli* (O26:B6) lipopolysaccharide (LPS), and polyinosinic:polycytidylic acid, a double-stranded RNA analog (cat no. I3036; S-poly(I:C), Supplementary Fig. 4a), were purchased from Sigma-Aldrich. Unless otherwise specified, S-poly(I:C) was used to induce IFN-β production. Another poly(I:C) (cat no. ALX-746-021), which was termed L-poly(I:C) (Supplementary Fig. 3a), was purchased from ENZO Life science (New York, NY, USA). HCV 3′-untranslated region (UTR) RNA containing 5′-end triphosphate and poly-U/UC region was prepared by in vitro transcription using T7 MEGAscript kit (Ambion, TX, USA) and a template amplified from pZS2 plasmid (kindly provided by Dr. Christoph Seeger, Institute for Cancer Research, Fox Chase Cancer Center, Philadelphia, PA, USA), an HCV subgenomic replicon derived from the parental HCV Con-1 replicon I_377_/NS3-3′ (AJ242652) by PCR using a set of primers [forward primer, 5′-TAATACGACTCACTATAGGTAGGGGTAGGCATCTATCTA-3′ (T7 promoter: underlined) and reverse primer, 5′-ACATGATCTGCAGAGAGGCC-3′]. The siRNAs for RIG-I, MDA5, and MAVS targeted the following sequences: siRIG-I, 5′-GAATTTAAAACCAGAATTATC-3′; siMDA5, 5′-GTGCATGAGGGAGGAACTG-3′; and siMAVS, 5′-AGGAGAGAATTCAGAGCAA-3′. Antibodies were obtained as follows: mouse monoclonal anti-MDA5 (C-5; 1:1,000 dilution), mouse monoclonal anti-MAVS (E-3; 1:1,000 dilution), mouse polyclonal anti-IRF-3 (FL-425; 1:1,000 dilution), and mouse monoclonal anti-human CD81 (5A6; 1:1,000 dilution) antibodies from Santa Cruz Biotechnology (Santa Cruz, CA, USA); mouse monoclonal anti-HA-tag (6E2; 1:1,000 dilution), rabbit monoclonal anti-phospho-IRF-3(Ser396; 1:1,000 dilution), rabbit monoclonal anti-phospho-TBK1(Ser172) (D52C2, 1:1,000 dilution), rabbit polyclonal anti-TBK1 (#3013; 1:1,000 dilution), rabbit polyclonal anti-TRIM25 (#12315; 1:1,000 dilution), and rabbit polyclonal anti-β-actin (#4967; 1:5,000 dilution) antibodies from Cell Signaling Technology (Beverly, MA, USA); rabbit polyclonal anti-RIG-I (ab45428;1:1,000 dilution), anti-Riplet (ab28636; 1:1,000 dilution), and anti-VP1 (norovirus capsid protein, ab92976; 1:1,000 dilution) antibodies from Abcam (Cambridge, UK); mouse monoclonal anti-α-tubulin antibody (DM1A; 1:5,000 dilution) from Calbiochem (La Jolla, CA, USA); rabbit polyclonal (F7425; 1:500 dilution for immunoprecipitation or 1:1,000 dilution for immunoblotting) or mouse monoclonal (M2; 1:1,000 dilution for immunoblotting) anti-Flag epitope antibodies from Sigma-Aldrich; and mouse monoclonal anti-Lamin A/C antibody (clone 14/LaminAC; 1:1,000 dilution) from BD Bioscience (Franklin Lakes, NJ, USA). [^14^C]Cyclo(Phe-Pro) with specific activity of 2.16 GBq mmol^−1^ (58.3 mCi mmol^−1^) was synthesized by Curachem (Cheongju, Korea).

### Plasmids transfection in vivo siRNA delivery

The pJFH1 plasmid^59^, which was used to produce infectious HCV (genotype 2a) particles, was provided by Dr. Takaji Wakita (National Institute of Infectious Diseases, Kyoto,  Japan). The pJFH1/GFP^60^ harboring a GFP-coding gene in the context of genotype 2a full-length HCV (JFH1) cDNA clone was provided by Dr. Xulin Chen (Institute for Virus Research, Chinese Academic of Sciences, Wuhan, China). The pCS4-3xHA-Ub plasmid^61^, which expresses a HA-tagged ubiquitin, was provided by Prof. Jong-Bok Yoon (Yonsei University, Seoul, Korea). Plasmids expressing hTLR4, MD2, and CD14^62^ individually were provided by Prof. Seung Hyun Han (Dental Research Institute, School of Dentistry, Seoul National University, Seoul, Korea). The reporter plasmid pRL-TK, which was used as an internal control for transfection efficiency, was obtained from Promega (Madison, WI, USA). The pFlag-CMV4-RIG-I plasmid was used to express wild-type human RIG-I (1–925 amino acids) in cells. The phRIG-I-CARD-Flag and pmRIG-I-CARD-Flag carrying a cDNA for the N-terminal two CARD-domain of human (1–284 amino acids) and mouse (1–285 amino acids) RIG-I, respectively, in the pcDNA3.1 expression vector (Invitrogen, Carlsbad, CA, USA) were used to express the C-terminal Flag-epitope-tagged RIG-I 2CARD domain. To overexpress MAVS protein, pFlag-hMAVS plasmid was constructed by inserting the MAVS cDNA (RefSeq Accession NM_001206491), which was amplified by RT-PCR using forward (5′-AAGCTTACCATGCCGTTTGCTGAAGAC-3′) and reverse (5′-TCTAGACTAGTGCAGACGCCGCCGG-3′) primers, into HindIII and XbaI sites of pcDNA3.1 vector (Invitrogen). All plasmids were transfected into cells using Fugene HD transfection reagent (Roche Applied Science, IN, USA) or Lipofectamin 2000 (Invitrogen). pTrcHisB-RIG-I was used to express an N-terminal (His)_6_-tagged, full-length recombinant RIG-I in *E. coli*.

HCV genomic RNA was transfected into Huh7 or mouse primary hepatocytes using *Trans*IT mRNA transfection reagent (Mirus Bio LLC, Madison, WI, USA). Poly(I:C), HCV 3′-UTR RNA, and siRNA were transfected into cells using Lipofectamine RNAiMAX (Invitrogen). Poly(I:C) was intravenously delivered into mouse liver tissues using the lipidoid ND98 (98N12-5) reagent. The lipidoid-RNA complexes encapsulated within nanoparticles were prepared as described with slight modifications^41^. Briefly, stock solutions of ND98, polyethylene glycol (PEG) 2000-ceramide C16 (Avanti Polar Lipids, AL, USA), and cholesterol (Sigma-Aldrich), which were all prepared in ethanol, were mixed at a molar ratio of 42:10:48. The mixture was dropwise added to 50 mM sodium acetate buffer (pH 5.2) to yield a solution of mixed lipids in 35% ethanol, resulting in formation of empty lipidoid nanoparticles. After extruding the resulting nanoparticles several times through polycarbonate filter membrane with 100 nm diameter pores using an Avanti Mini-Extruder apparatus (Avanti Polar Lipids Inc., Alabaster, AL, USA), poly(I:C) (10 mg ml^−1^) in 35% ethanol and 50 mM sodium acetate (pH 5.2) was then added to the nanoparticles at 1:7.5 (wt/wt) poly(I:C)/total lipids. After incubation at 37 °C for 30 min, poly(I:C)-containing lipidoid nanoparticles were subjected to dialysis against PBS using a Slide-A-Lyzer dialysis cassette (molecular cut-off of 3.5 kDa) (Thermo Scientific, Rockford, IL, USA) to remove ethanol and unencapsulated poly(I:C). The RNA quantity within the nanoparticles was determined by the Quant-iT RiboGreen RNA assay (Invitrogen).

### Cell lines and culture

The human hepatocellular carcinoma Huh7 cell line and Huh7.5.1, a highly HCV-permissive Huh7.5-derived cell line due to loss of RIG-I function^29^ (kindly provided by Dr. Frank Chisari, Scripps Research Institute, La Jolla, CA, USA) were cultivated in Dulbecco’s modified Eagle’s medium (DMEM) supplemented with 10% fetal bovine serum, 2 mM L-glutamine, 100 U ml^−1^ of penicillin, 100 μg ml^−1^ streptomycin and 0.1 mM nonessential amino acids at 37 °C in 5% CO_2_. The murine macrophage cell line RAW264.7 and human embryonic kidney 293T (HEK293T) cell line were cultivated in DMEM supplemented with 10% FBS, 100 U ml^−1^ of penicillin, and 100 μg ml^−1^ streptomycin. Huh7, HEK293T (CRL-3216), and RAW264.7 (TIB-71) cells were purchased from American Type Culture Collection (ATCC; Rockville, MD, USA). Normal human primary hepatocytes (HH, #5200), which were prepared from liver tissues obtained from patients with informed consent from donor or donor’s family aged over 18 years, were purchased from ScienCell Research Laboratories (Carlsbad, CA, USA) and cultivated according to manufacturer′s recommendations. Mouse primary hepatocytes were isolated using an in situ two-step perfusion method and cultivated in HCM media on collagen-coated culture plates, as described previously^42^.

### Establishment of MAVS knockout Huh7

Huh7 cells were transfected with a mixture of three CRISPR-Cas9-GFP expressing plasmids that individually express a different MAVS-targeting guide RNA (sc-400769, Santa Cruz, CA, USA). GFP-positive cells were selected according to the manufacturer’s instructions. For gene KO analysis by sequencing, genomic DNA was extracted with the Wizard Genomic DNA purification kit (Promega), and the edited gene loci were amplified by PCR using appropriate primer sets for each guide RNA-targeting site. The resulting PCR product was cloned into pCR2.1-Topo vector (Invitrogen) for sequence analysis.

### Viruses and plaque-forming assay

HCV (JFH1, AB047639) RNA was prepared by T7 RNA polymerase-mediated in vitro transcription and electroporated into Huh7 cells, as described previously^42^. Infectious particles in the culture medium were collected by centrifugation and were used for infection experiments, as described previously^63^. Mouse sera, which were derived from HCV genotype (GT) 1b (patient sera)-infected uPA^+/+^SCID^+/+^ mice transplanted with human hepatocytes, were obtained from Phoenix Bio (Hiroshima, Japan) and used to infect primary human hepatocytes. Murine norovirus 1 (MNV-1) strain CW1 kindly provided by Prof. Herbert Virgin IV (Washington University School of Medicine, St. Louis, MO, USA) was propagated in RAW264.7 cells, as described previously^64^. Sendai virus (SeV) (Cantell strain; ATCC VR-907) used in infection experiments was propagated in Huh7 cells following a protocol described previously^65^. SeV stock used for mouse infection was prepared by inoculation of 10-day embryonated chicken eggs, as described^66^. Influenza A/PR8 (H1N1) virus, a mouse-adapted strain (kindly provided by Prof. Heung Kyu Lee, KAIST, Daejeon, Korea), was cultivated, as described previously^67^. Plaque forming assays for MNV-1, SeV, and influenza virus were performed using RAW264.7, Vero E6, and MDCK cells, respectively, as reported previously^33,66,67^.

### Real-time reverse-transcription quantitative PCR

Total RNA from cells or mouse serum was extracted with TRIzol or TRIzol LS reagent (Invitrogen), respectively. HCV plus-sense (genomic RNA) and minus-sense RNA levels were quantified using a TaqMan probe specific for the HCV 5′-UTR region, as described previously^42^. MNV-1 genomic copy number was measured by the TaqMan probe quantification assay^61^. Real-time mRNA quantification for IFN-α, IFN-β, IFN-stimulated gene 56 (ISG56), 2′-5′-oligoadenylate synthetase (2′-5′ OAS), and glyceraldehyde 3-phosphate dehydrogenase (GAPDH) was performed using SYBR Premix ExTaq (Takara). SeV genome titer was determined by RT-qPCR using specific sets of primers, as described^66^, using SYBR Premix ExTaq (Takara). All target gene levels normalized with GAPDH mRNA level were quantified using the ΔΔ*C*_t_ method^68^. The primers used for mRNA quantification were previously reported as follows: human IFN-β^69^ and human ISG56^70^, human 2′-5′ OAS^71^, and mouse IFN-α and β^72^.

### Cell viability

Huh7 cells were split into a 96-well plate (2 × 10^4^ cells per well) and treated with various concentrations of cFP for 48 h. Cell viability was measured using an MTS [3-(4,5-dimethylthiazol-2-yl)-5-(3-carboxymethoxyphenyl)-2-(4-sulfophenyl)-2H-tetrazolium] assay (Promega) according to the manufacturer’s protocol. The IC_50_ values were calculated using SigmaPlot software (Systat Software Inc., Hounslow, London, UK)

### Fluorescence microscopy

JFH1/GFP-transfected Huh7 cells grown on Lab-Tek 4-well chamber slides (Nunc, Roskilde, Denmark) were fixed with 4% paraformaldehyde (Sigma-Aldrich) for 15 min. After washing three times with PBS, nuclei were visualized by staining with 1 μM 4′,6′-diamidino-2-phenylindole (DAPI). Fluorescent signal was visualized using a confocal laser scanning microscope (Zeiss LSM 510 META, Carl Zeiss, Oberkochen, Germany). The GFP-positive area among 500 total cells was quantified using ImageJ program (https://imagej.nih.gov/ij/).

### Immunoblotting and immunoprecipitation

Cells were lysed in a lysis buffer (50 mM Tris–HCl, pH 7.5, 150 mM NaCl, 1 mM EDTA, 1% Nonidet P-40, 50 mM NaF, 1 mM tetrasodium pyrophosphate, 17.5 mM β-glycerophosphate, and 1 mM Na_3_VO_4_) supplemented with an EDTA-free protease inhibitor cocktail (Roche Diagnostics, Mannheim, Germany) by incubating on ice for 20 min. Cleared cell lysates were resolved by sodium dodecyl sulfate polyacrylamide gel electrophoresis (SDS–PAGE), followed by immunoblot analysis using appropriate antibody sets and the enhanced chemiluminescence kit (ECL, GE Healthcare Life Sciences, Piscataway, NJ, USA). To detect IRF-3 dimers, protein samples were subjected to electrophoresis on a native polyacrylamide gel, as previously described^62^. Endogenous RIG-I and epitope-tagged transiently expressed proteins were immunoprecipitated using protein G-coated magnetic beads (Magnetic Dynabeads-Protein G; Invitrogen). Uncropped images of immunoblots are provided in Supplementary Figs. 9–11.

### cFP uptake assay

[^14^C]cFP (10 µM) in complete media was incubated with Huh7 cells at 37 °C for 5, 15, 30, or 60 min at 37 °C. After washing cells twice with ice-cold PBS and lysing in the cell lysis buffer described above, radioactivity of lysates was counted in a Wallac 1409 liquid scintillation counter (Perkin Elmer, Waltham, MA, USA). When indicated, cells were fractionated, as described below prior to measuring the [^14^C]cFP levels.

### Subcellular fractionation

Subcellular fractions of Huh7 cells were isolated by differential centrifugation, as reported previously^73^ with slight modifications. After washing cell pellets with buffer A (20 mM MOPS, pH 7.4, 100 mM sucrose, and 1 mM EGTA), cells were lyzed in buffer B [buffer A plus 5% Percoll (Sigma-Aldrich), 0.01% digitonin, and protease inhibitor cocktail (Roche)] by incubation on ice for 20 min. Crude nuclear fraction was collected by centrifugation at 2500 × *g* for 5 min following washing the nuclear pellet once with buffer B. The nuclear fraction resuspended in buffer C (10 mM Tris–HCl, pH 7.5, 2.5 mM KCl, and 2.5 mM MgCl_2_) was then centrifugated at 90,000 × *g* for 30 min through 2.1 M sucrose in 50 mM Tris–HCl, pH 7.5, and 5 mM MgCl_2_ to obtain pure nuclear fraction. The nucleus-cleared supernatant collected by centrifugation at 2500 × *g* was subjected to centrifugation at 15,000 × *g* for 15 min to obtain the P15 fraction enriched with mitochondria. The recovered supernatant was then further centrifugated at 100,000 × *g* for 1 h. The resultant supernatant and the pellet were designated the S100 fraction representing soluble cytoplasmic fraction and the P100 fraction enriched with microsomes and cellular membranes, respectively.

### In vitro kinase assay

In vitro kinase assay with TBK1 was performed, as previously described^74^. Briefly, TBK1 in HEK293T total cell lysate was immunoprecipitated using a specific antibody immobilized to Dynabeads-Protein G (Invitrogen). Immunocomplexes were washed three times in the cell lysis buffer described above and twice in a reaction buffer (50 mM Tris–HCl, pH 7.5, 0.1% β-mercaptoethanol, 0.1 mM EGTA, and 10 mM magnesium acetate), and then resuspended in the 50-μl reaction buffer with or without cFP. The reaction was initiated by the addition of 10 μCi of [γ-^32^P]-ATP and terminated by boiling at 95 °C after 1 h incubation at 30 °C. Protein samples were then subjected to SDS-8% PAGE for subsequent autoradiography.

### Expression and purification of RIG-I

Expression of RIG-I in *E. coli* BL21 was induced with 0.5 mM IPTG for 20 h at 16 °C. Soluble RIG-I protein was then purified using Ni-nitrilotriacetic acid-Sepharose resin (Qiagen, Hilden, Germany) pre-equilibrated with the binding buffer (50 mM Na-phosphate, pH 7.8, 300 mM NaCl, 10 mM imidazole, 10 mM β-mercaptoethanol, 1% Nonidet P-40, and 10% glycerol). After washing several times in the binding buffer, bound proteins were eluted with 100 mM imidazole. The eluted fraction dialyzed against buffer A (50 mM Tris–HCl, pH 7.8, 1 mM dithiothreitol, 50 mM NaCl, 5 mM MgCl_2_, and 10% glycerol) was further purified using a Q-Sepharose column (GE Healthcare, Uppsala, Sweden) for anion exchange chromatography, and a heparin Sepharose column (GE Healthcare). Eluted recombinant RIG-I proteins were dialyzed against buffer A and stored at −80 °C in small aliquots.

### Pull-down experiments

HEK293T cell lysate from two 10-cm plates was mixed with 1 × 10^13^ copies of biotinyl cFP for 2 h at 4 °C on an orbital rotator. After 2 h binding, 50-μl of the streptavidin-coated magnetic beads (Dynabeads M-280; Invitrogen) pre-equilibrated with the cell lysis buffer was added to the cell lysate-cFP mixtures and incubated further for 30 min. When indicated, alternatively, cFP-linked Sepharose beads were used in pull-down experiments. Bead-protein complexes were washed three times with 10-ml cell lysis buffer and then the pull-down proteins were denatured by boiling in SDS sample buffer, then analyzed by immunoblotting. To pull-down endogenous RIG-I using in vitro transcripts of HCV 3′-UTR RNA, 5′-biotinyl PNA-SL3-17 (5′-biotin-OO-GCTAAGATGGAGCCACC-3′; Panagene, Daejeon, Korea), which hybridizes to stem-loop III of the X-tail region of HCV 3′-UTR, was used. HEK293T cell lysates from 2 × 10-cm dishes were mixed with 1 × 10^12^ copies of HCV 3′-UTR RNA, 1 × 10^13^ copies of 5′-biotinyl PNA-SL3-17, RNase inhibitor (4 U ml^−1^, Promega), and various amounts of cFP. After incubation at 4 °C on an orbital rotator for 2 h, immunoblotting analyses were performed, as described above.

### Limited trypsin digestion of RIG-I

To carry out trypsin proteolysis, 400 ng of purified RIG-I with or without cFP (1 mM) was mixed with 1 μg of HCV 3′-UTR RNA or poly(I:C) in a 20-μl reaction buffer (20 mM Tris–HCl, pH 8.0, 1.5 mM MgCl_2,_ 1.5 mM DTT, and 70 mM KCl) supplemented with 6.7 µg of AMP-PNP (Sigma). After 5 min incubation at room temperature, sequencing grade TPCK-treated modified trypsin (Promega) was added to the reaction mixture at a RIG-I:trypsin ratio of 100:1 (w/w) and further incubated at 37 °C. The reaction was terminated by addition of 0.5 μg of N-α-Tosyl-L-lysine chloromethyl ketone hydrochloride (TLCK) (Sigma), which is an irreversible inhibitor of trypsin. After 5 min incubation at room temperature, protein samples were subjected to SDS-14% PAGE followed by immunoblotting analysis using a RIG-I-specific antibody.

### Animal experiments

BALB/cCrSlc, non-obese diabetic/severe combined immunodeficient (NOD/SCID; substrain NOD.CB17-PrkdcSCID/ARC), and DBA/2CrSlc mice were purchased from Central Lab Animal Inc. (Seoul, Korea). C57BL/6NCrljOri mice were purchased from Orient Bio (Seongnam, Korea). For isolation of mouse primary hepatocytes, BALB/c male mice (8 weeks of age) were anesthetized with avertin (intraperitoneally) and perfused via the portal vein into liver tissues with warmed EGTA solution (37 °C) at a flow rate of 5 ml min^−1^ for 5 min. Then, livers were perfused with collagenase in the same solution (37 °C) supplemented with 5 mM CaCl_2_ and 50 mM HEPES at a flow rate of 5 ml min^−1^ for 5 min. After dispersal and filtering of cells from perfused liver tissues, hepatocytes were separated by centrifugation at 50 × *g* for 5 min. Hepatocytes from MAVS knockout mice (B6;129-*Mavs*^*tm1Zjc*^/J)^20^, which were obtained from the Jackson Laboratory (stock No: 008634; Bar Harbor, ME, USA), were also similarly prepared.

To evaluate in vivo efficacy of cFP on type I IFN induction, BALB/c male mice (8 weeks of age, 20–23 g body weight) were given ND98-formulated lipid nanoparticles carrying poly(I:C) (1 mg per kg body weight), and dipeptides dissolved in 100 μl of saline (50 mg per kg body weight) through the tail vein. After 8 h, large lobes of the liver were retrieved and homogenized to extract total RNA using TRIzol reagent.

To evaluate in vivo efficacy of cFP on HCV, NOD/SCID mice bearing HCV-replicating Huh7 xenografts were used^42^. HCV RNA-transfected cells mixed with Matrigel were injected subcutaneously or into the large lobes of the livers of anesthetized immunodeficient NOD/SCID male mice (5 weeks of age, 18–20 g body weight). Four weeks after implantation, dipeptides (50 mg per kg body weight) in 100-μl of saline were given by i.v. injection following administration of poly(I:C) (1 mg per kg body weight) formulated within lipidoid nanoparticles^42^. Serum HCV titer was monitored by RT-qPCR. DBA/2 mice (6-week-old male, 15–17 g body weight) were used for SeV infection experiments. Mice were infected intranasally with 10^5^ PFU of SeV in a volume of 50-μl saline. Survival and weight loss were monitored daily for 10 days. Whole lungs of SeV-infected mice were harvested at day 7 post-infection. The lung tissue of each mouse was divided into two parts; one was used for virus titration and the other was processed for H&E staining. Lung tissues were homogenized in 1 ml PBS and centrifuged to harvest supernatants. Virus titers were determined by plaque assays and RT-qPCR. To evaluate in vivo effects of cFP on influenza infection, dipeptides (50 mg per kg body weight) were given to C57BL/6 mice (9-week-old male) via i.v. injection prior to intranasal influenza A/PR8 (H1N1) (1000 PFU) infection. In morbidity experiments, mice weight gain/loss was monitored daily and euthanized when their weight loss exceeded 20% of the average weight of age-matched control mice. Mice with this degree of weight loss showed signs of morbidity such as respiratory distress, lack of grooming with ruffled fur, and lethargy. All mice were housed and maintained in accordance with the relevant national and intuitional guidelines, and all mouse infection experiments were performed in approved ABSL-2 facilities.

### Ethics statement

All animal experiments were performed in accordance with the Korean Food and Drug Administration guidelines. Protocols were reviewed and approved by the Institutional Animal Care and Use Committees (IACUCs) of the Yonsei University (A-201408-274-01 and A-201712-474-01) and the Korea Research Institute of Chemical Technology (2017-8A-12-01). At the termination of experiments, all mice were euthanized by CO_2_ inhalation. The protocol for the uPA^+/+^SCID^+/+^ mouse infection experiments was approved by the local IACUC (Hiroshima, Japan).

### Statistical analysis

Results are presented as the mean±standard deviation (s.d.) from at least three independent experiments, unless otherwise stated. Statistical analyses (Student’s *t*-test) were performed using GraphPad Prism 6 (GraphPad Software Inc., La Jolla, CA, USA). Survival curves were analyzed using log rank (Mantel–Cox) test. Differences between groups were considered statistically significant at *P* < 0.05.

### Data availability

The authors declare that all data are available from the authors upon request.

## Electronic supplementary material

Supplementary Information
