# Supplementary material for: Vibrio vulnificus quorum-sensing molecule cyclo(Phe-Pro) inhibits RIG-I-mediated antiviral innate immunity
Source: Nat Commun. 2018 Apr 23;9:1606. doi: 10.1038/s41467-018-04075-1 (PMC5913291; doi:10.1038/s41467-018-04075-1)
Supplement: Supplementary file 1 — Supplementary Information [file 41467_2018_4075_MOESM1_ESM.pdf]

Supplementary Information

Vibrio vulnificus quorum-sensing molecule cyclo(Phe-Pro) inhibits RIG-I-mediated antiviral innate immunity

Wooseong Lee, Seung-Hoon Lee, Minwoo Kim, Jae-Su Moon, Geon-Woo Kim, Hae-Gwang Jung, In Hwang Kim, Ji Eun Oh, Hi Eun Jung, Heung Kyu Lee, Keun Bon Ku, Dae-Gyun Ahn, Seong-Jun Kim, Kun-Soo Kim & Jong-Won Oh*

Contents:

Supplementary Figures 1–11

Correspondence should be addressed to J.-W.O. (jwoh@yonsei.ac.kr).

Supplementary Figures

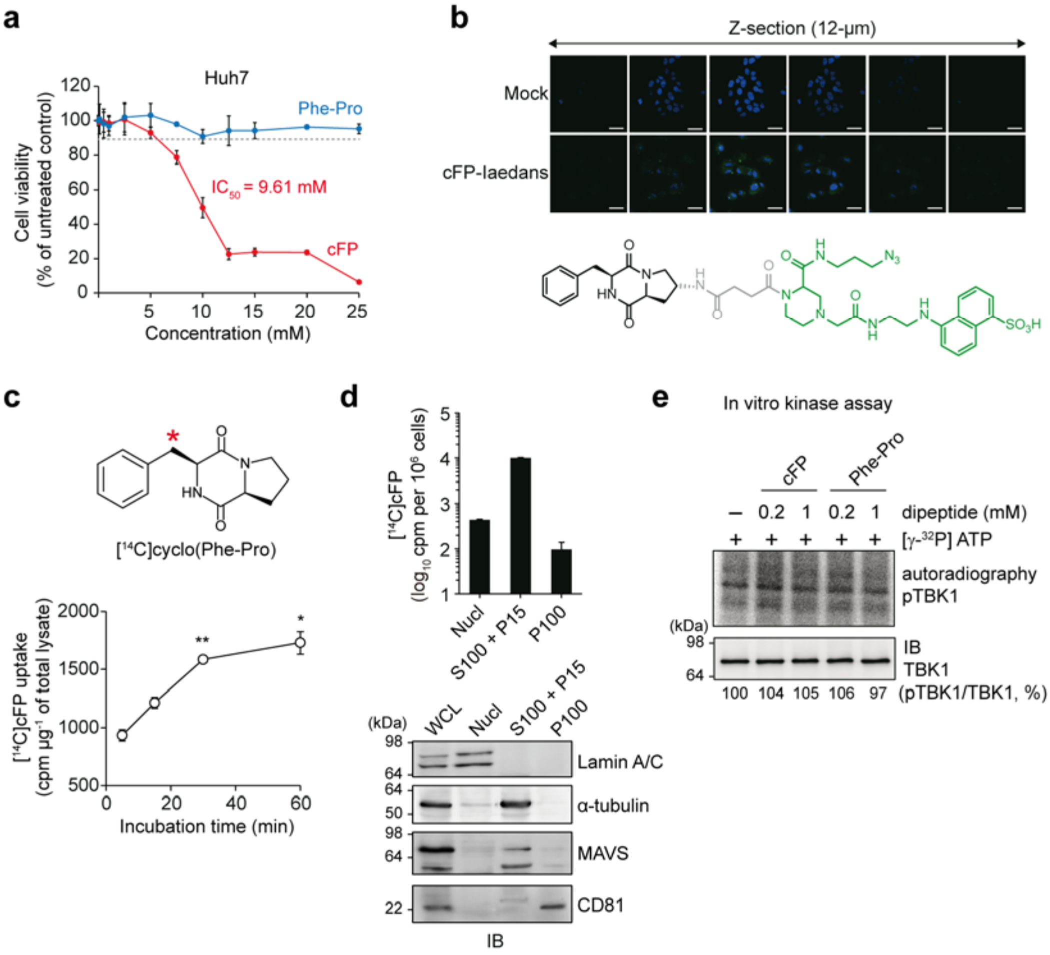

Supplementary Figure 1. (**a**) Cytotoxicity of cFP in Huh7 was assessed two days after cFP treatment. **(b)** Cellular entry of cFP. Huh7 cells grown in a chamber slide were incubated with 20 mM IAEDANS-labeled cFP for 60 min. After washing three times with PBS, cells were fixed in 4% paraformaldehyde solution. Z-section images were taken with a confocal microscope. Scale bar, 50 µm. (**c**) Intracellular [^{14}C]cFP levels were measured following incubation of Huh7 cells with 10 µM [^{14}C]cFP for the indicated time periods. Data are mean ± s.d. **P* < 0.05 and ***P* < 0.01 (Student's *t*-test) compared to radioactivity measured after 5 min incubation. (**d**) Huh7 cells incubated with 10 µM [^{14}C]cFP for 60 min were subjected to cellular fractionation prior to measuring the radioactivity in the indicated subcellular fractions as in (**c**). Indicated fractions derived from an equal cell number were resolved by SDS-PAGE for immunoblotting (IB) analysis. Nucl, nuclear fraction; S100 + P15, soluble cytoplasmic fraction plus the fraction enriched with mitochondria; P100, fraction enriched with microsomes and cellular membranes; WCL, whole cell lysate. Lamin A/C, a nuclear marker; α-tubulin, a cytoplasmic fraction marker, MAVS, mitochondrial antiviral-signaling protein; CD81, an HCV receptor. (**e**) *In vitro* kinase assays were carried out using immunoprecipitated TBK1 in the absence or presence of increasing concentrations of cFP or Phe-Pro. Phosphorylated TBK1 was visualized by autoradiography.

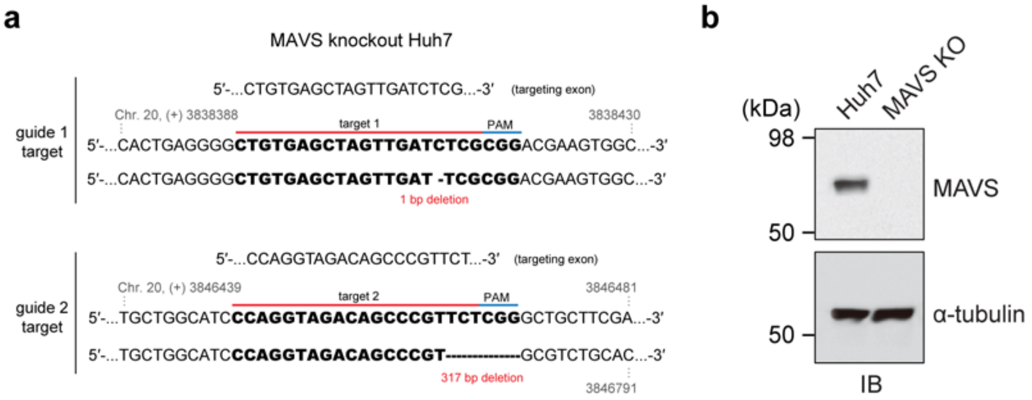

Supplementary Figure 2. Depletion of MAVS expression in Huh7 cells by CRISPR/Cas9-directed genome editing. (**a**) Establishment of a Huh7-derived MAVS KO cell line. Sequence analyses of MAVS gene edited by the two different guide RNAs targeting MAVS exon sequences. (**b**) Immunoblotting analysis of MAVS depletion in MAVS KO Huh7 cells.

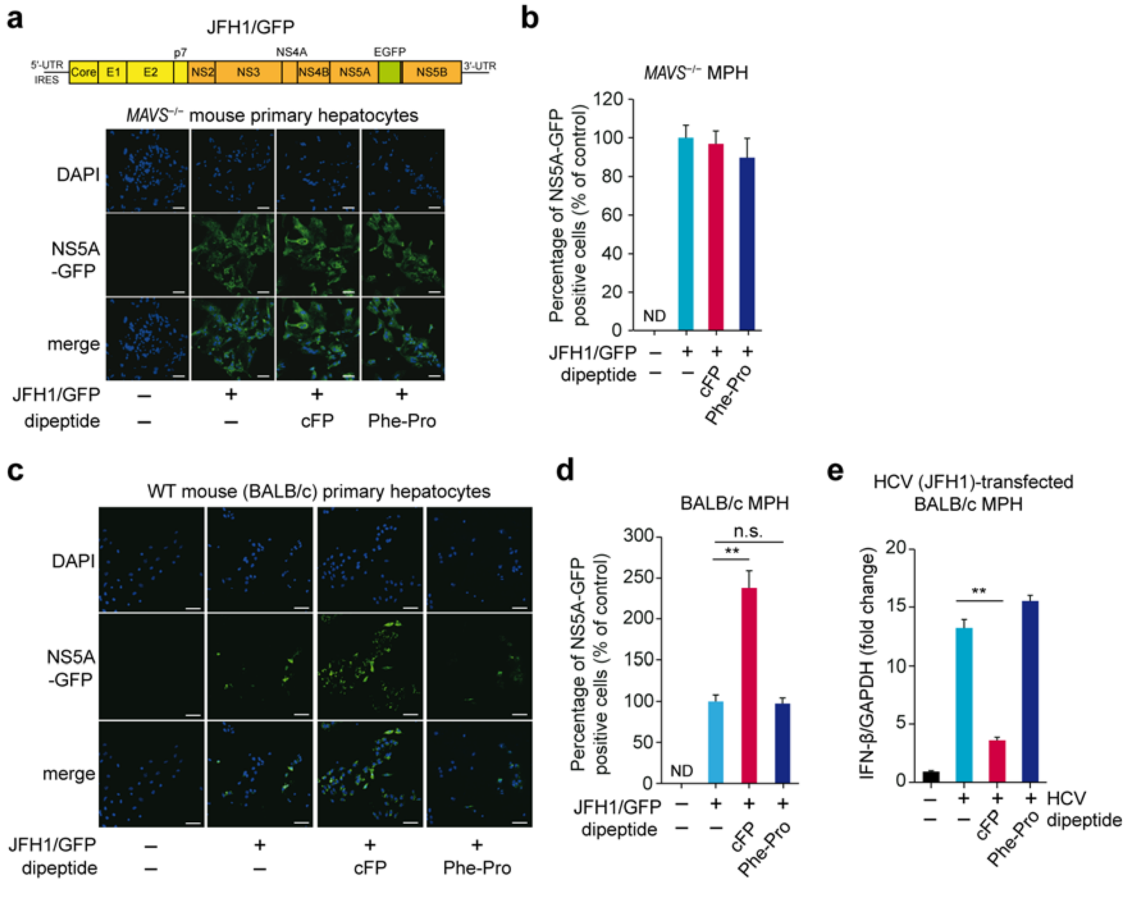

Supplementary Figure 3. MAVS-dependent regulation of HCV by cFP. Primary hepatocytes from *MAVS*$^{-/-}$ (**a**–**b**) or BALB/c mice (**c**–**e**) were transfected with HCV JFH1/GFP (**a**–**d**) or JFH1 RNA (*in vitro* transcripts) and treated with 2.5 mM cFP or Phe-Pro for 48 h, prior to confocal microscopic analysis (**a**,**c**), and quantification of GFP fluorescence intensity (**b**,**d**) and IFN-β mRNA levels (**e**). Scale bar, 100 μm. MPH, mouse primary hepatocytes. Data are mean ± s.d. Statistical significance of differences between groups was determined via unpaired two-tailed Student's *t*-test. ***P* < 0.01; n.s., not significant.

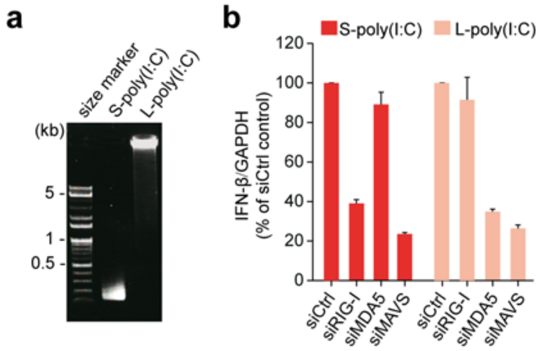

Supplementary Figure 4. Differential regulation of IFN-β expression by poly(I:C) reagents of different lengths. (**a**) Analysis of poly(I:C) reagents by agarose gel electrophoresis. S-poly(I:C), short-length poly(I:C); L-poly(I:C), extended-length poly(I:C). (**b**) RT-qPCR quantification of IFN-β mRNA levels in HEK293T cells treated with the indicated siRNAs (30 nM each) for 24 h, followed by stimulation with the indicated poly(I:C) reagents at 2 μg ml^{-1} for 24 h.

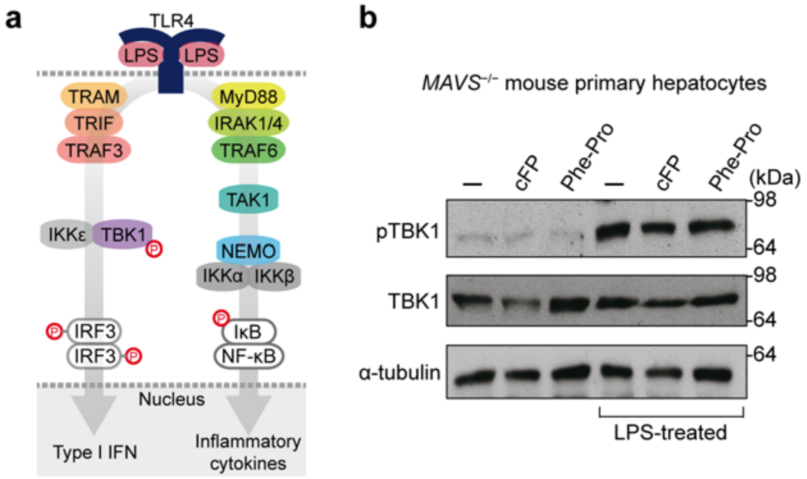

Supplementary Figure 5. cFP does not act on the TLR4-TRIF axis in TLR4 signaling pathways. (a) A simplified scheme of TLR4 signaling pathways. (**b**) *MAVS*$^{-/-}$ mouse primary hepatocytes, which were transfected with plasmids expressing TLR4, MD2, and CD14 individually, were stimulated with LPS (1 μg ml^{-1}) 12 h after plasmid transfection in the absence or presence of the indicated dipeptides (5 mM each). After 12 h, cell lysates were analyzed by immunoblotting for the indicated proteins.

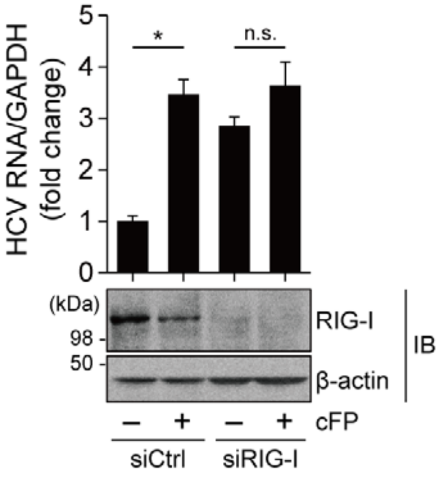

Supplementary Figure 6. HCV regulatory activity of cFP is mediated through RIG-I-MAVS axis. Huh7 cells, which were pre-treated with a control siRNA (siCtrl) or a RIG-I-specific siRNA (siRIG-I) for 12 h, were infected with HCV (JFH-1) at a MOI of 0.25 and treated with 2.5 mM cFP. After 36 h, cells were harvested for determination of HCV RNA titer and immunoblotting analysis for the indicated proteins. Statistical significance of differences between groups was determined via unpaired two-tailed *t*-test. **P* < 0.05; n.s., not significant.

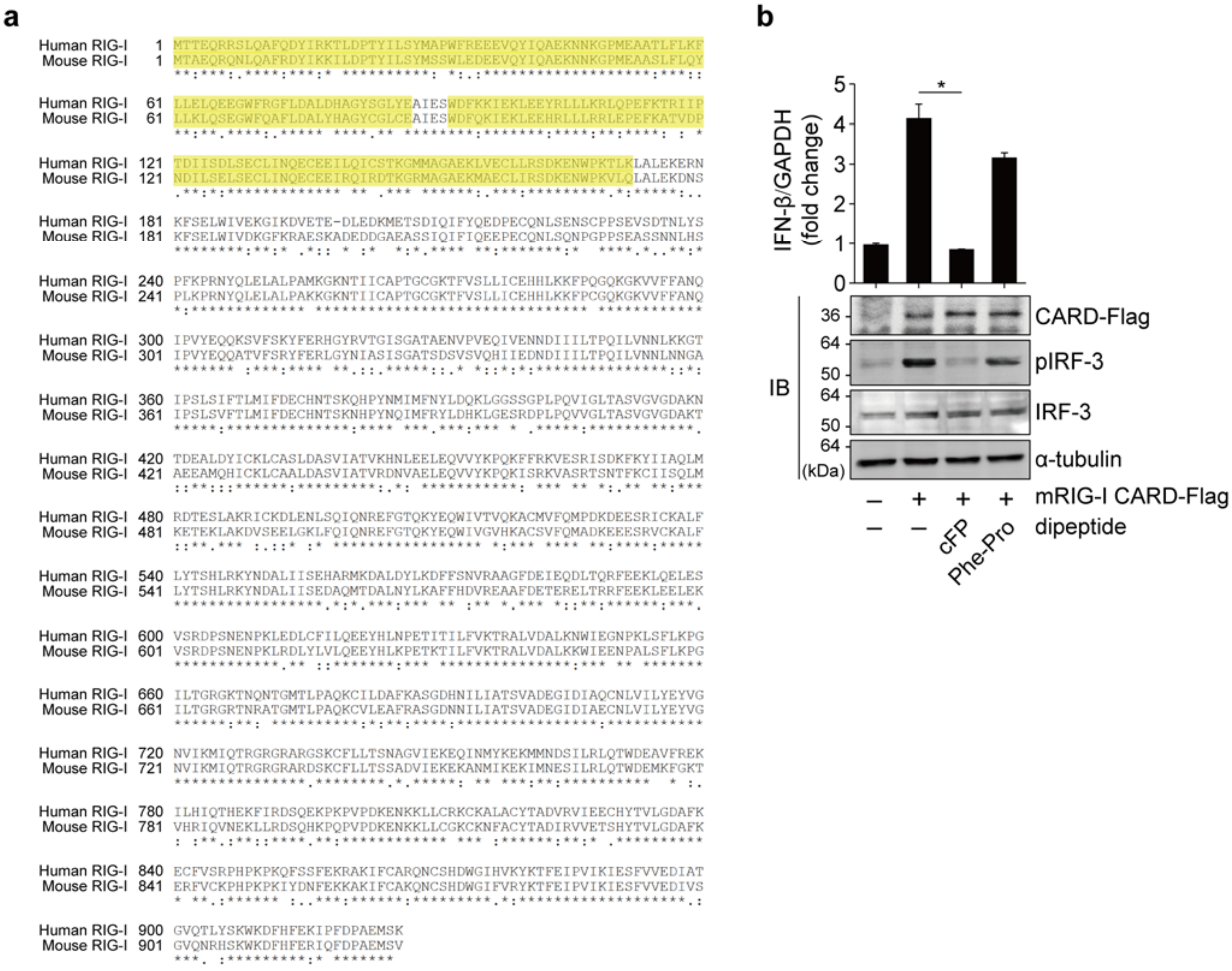

Supplementary Figure 7. Dysregulation of mouse RIG-I by cFP. (**a**) Alignment of human (UniProtKB: O95786) and mouse (UniProtKB: Q6Q899) RIG-I amino acid sequences using Clustal Omega program. "*", fully conserved residue; ":", strongly similar properties; ".", weakly similar properties. The N-terminal 2CARD domain (denoted by yellow boxes) shows 77% sequence identity between human and mouse RIG-I proteins. (**b**) HEK293T cells were transfected with an empty vector or a plasmid expressing the mouse Flag-tagged CARD domain (mRIG-I CARD-Flag). Six hour later, cells were treated with cFP or Phe-Pro (2.5 mM) for 30 h, prior to quantification of IFN-β mRNA levels and immunoblotting analysis for the indicated proteins. Statistical significance of differences between groups was determined via unpaired two-tailed *t*-test. *$P < 0.05$.

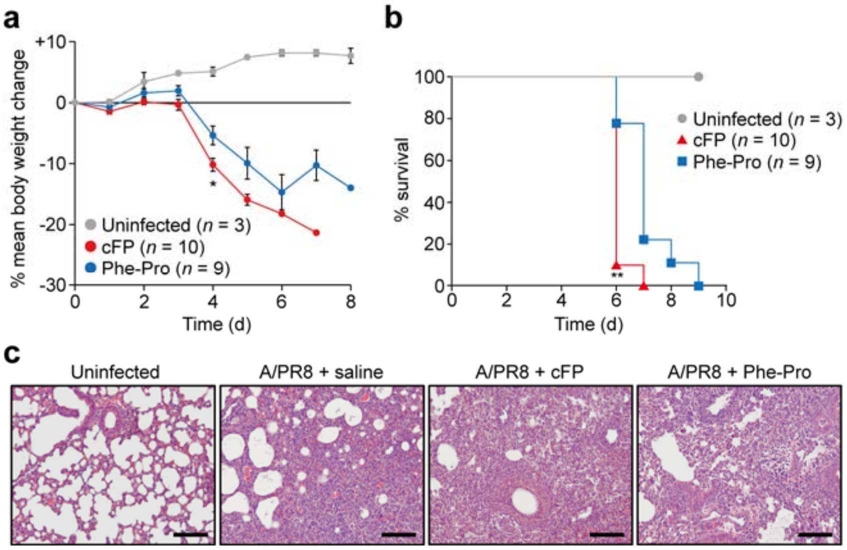

Supplementary Figure 8. Increase of mortality of influenza-infected mice by cFP administration. Wild-type C57BL/6 mice (9-week-old male), which were given cFP or Phe-Pro (50 mg kg^{-1} body weight) by i.v. injection, were infected with influenza virus (PR8 strain, 1,000 PFU) by intranasal injection two hours after the dipeptide administration followed by two additional consecutive administrations at 1 and 2 dpi, prior to monitoring the change in body weight (**a**) and mouse mortality (**b**). Shown in (**c**) are representative H&E stain images of lung sections at 3 dpi. Scale bar, 100 μm. Statistical significance of differences between cFP-treated group and vehicle-treated, uninfected control group was determined via unpaired two-tailed *t*-test. Survival curves were analyzed using log rank (Mantel-Cox) test. $*P < 0.05$; $**P < 0.01$.

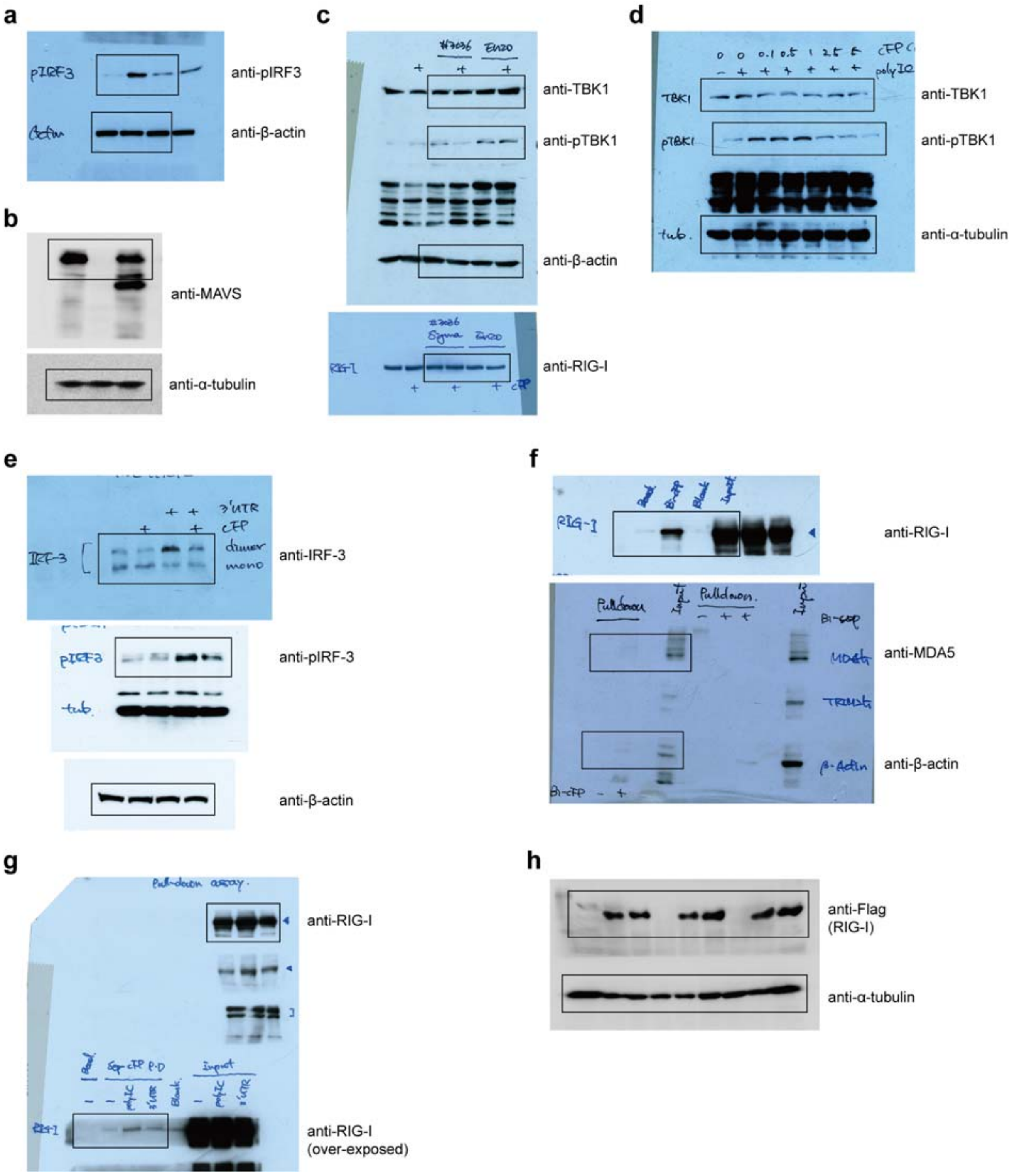

Supplementary Figure 9. Uncropped images of immunoblots for Figures 1–3. (**a**) For Fig. 1e. (**b**) For Fig. 1f. (**c**) For Fig. 2d. (**d**) For Fig. 2e. (**e**) For Fig. 2f. (**f**) For Fig. 3a. (**g**) For Fig. 3b. (**h**) For Fig. 3d.

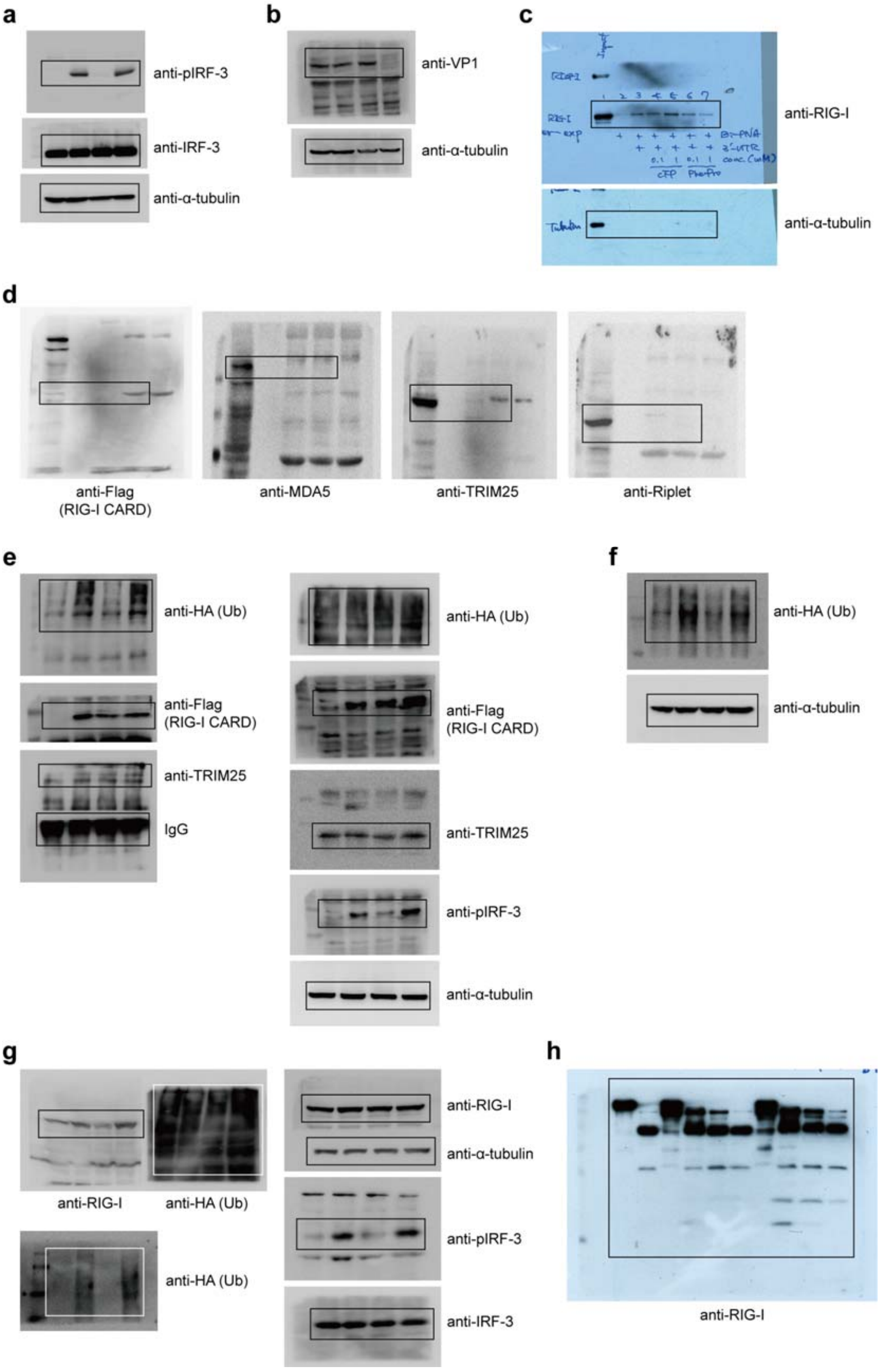

Supplementary Figure 10. Uncropped images of immunoblots. (**a**) For Fig. 4b. (**b**) For Fig. 4d. (**c**) For Fig. 5a. (**d**) For Fig. 5b. (**e**) For Fig. 5d. (**f**) For Fig. 5e. (**g**) For Fig. 5f. (**h**) For Fig. 5g.

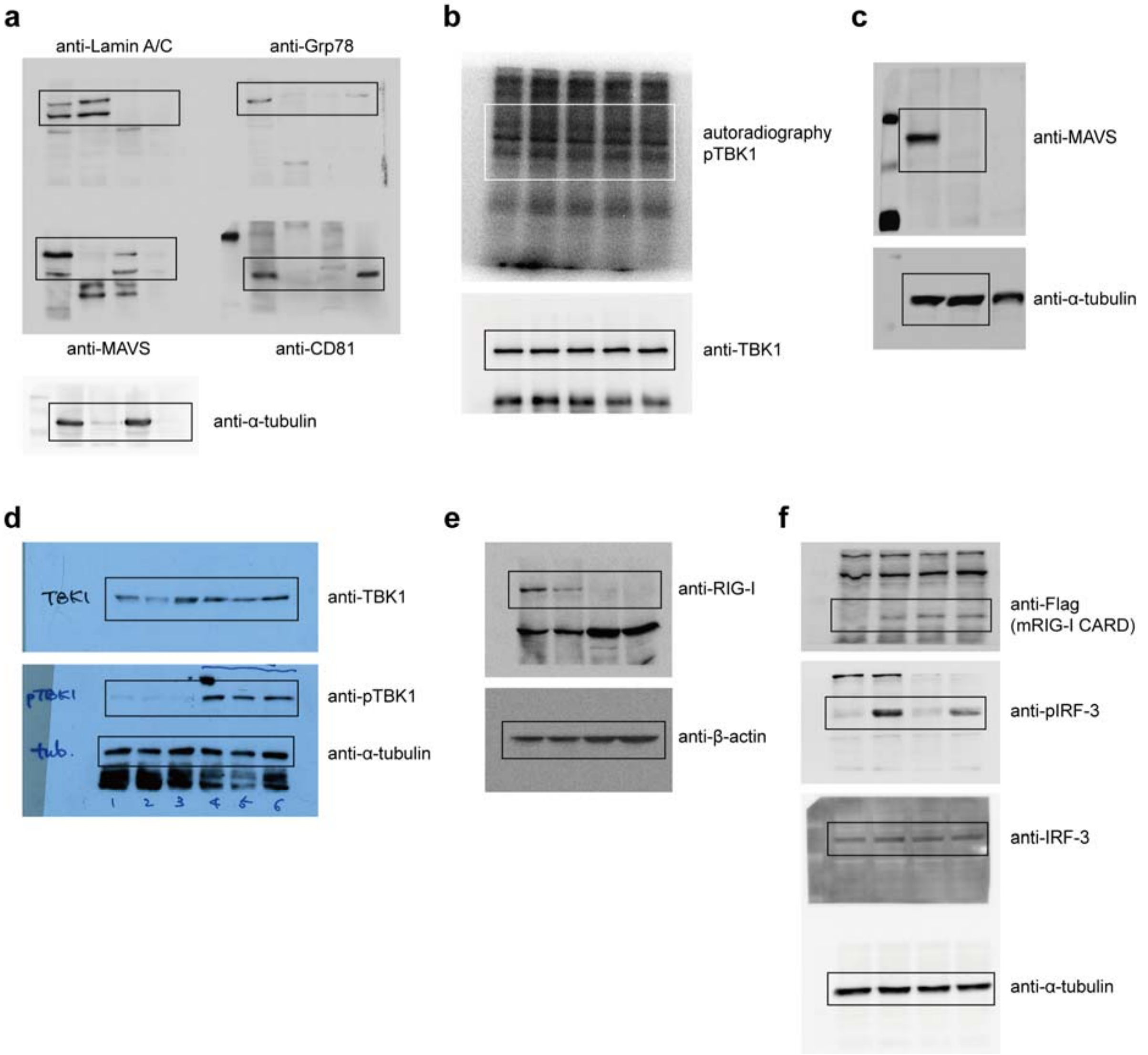

Supplementary Figure 11. Uncropped images of immunoblots. (**a**) For Supplementary Fig. 1d. (**b**) For Supplementary Fig. 1e. (**c**) For Supplementary Fig. 2b. (**d**) For Supplementary Fig. 5b. (**e**) For Supplementary Fig. 6. (**f**) For Supplementary Fig. 7b.
